# The Role of the Intestinal Microbiome in the Pathogenesis and Treatment of Hyperuricemia: A Review

**DOI:** 10.1002/fsn3.70982

**Published:** 2025-10-09

**Authors:** Junyu Yang, Jiali Chen, Zhenmin Liu, Yezhi Qu, Xiqing Yue, Bo Yuan, Mohan Li

**Affiliations:** ^1^ Department of Reparative and Reconstructive Surgery The Second Affiliated Hospital of Dalian Medical University Dalian China; ^2^ Shanghai Advanced Research Institute Chinese Academy of Sciences Shanghai China; ^3^ College of Food Science Shenyang Agricultural University Shenyang China; ^4^ State Key Laboratory of Dairy Biotechnology, Shanghai Engineering Research Center of Dairy Biotechnology Dairy Research Institute, Bright Dairy & Food Co., Ltd. Shanghai China; ^5^ School of Life Sciences & Biotechnology Shanghai Jiao Tong University Shanghai China

**Keywords:** gut microbiota, gut–kidney axis, hyperuricemia, probiotics, uric acid

## Abstract

Hyperuricemia (HUA), characterized by elevated blood uric acid (UA) levels, is a major risk factor for gout, UA nephropathy, metabolic syndrome, and other related disorders. Traditional drug therapy for HUA includes medications (e.g., allopurinol and febuxostat) and dietary changes; however, it is limited and may be accompanied by adverse side effects such as allergies, prompting the investigation of alternative therapeutic approaches. Although the newly researched “in‐situ graft polymerization” protein drug modification technology and the emerging gut microbiota transplantation technology have demonstrated innovation in regulating blood UA, they still need to overcome bottlenecks in immunogenicity, individual variability, and formulation technology. Recent research has highlighted the potential of modulating the intestinal microbiome as a promising strategy for managing HUA. Nevertheless, the mechanism by which different intestinal microbiomes affect HUA pathogenesis remains unclear. To bridge this gap, this review firstly outlines the characteristics and prevailing conditions of HUA, followed by the current status of treatment. Besides, this review integrates the findings from clinical trials and animal studies to explore in depth the pathogenic mechanisms of HUA and the potential roles and regulatory pathways of the gut microbiota in mitigating HUA. The gut microbiota act as multi‐functional factors that affect HUA by reducing UA production, enhancing purine metabolism, influencing amino acid transport, and increasing UA excretion. This review addresses critical gaps in the extant literature regarding microbiota‐mediated UA homeostasis and provides new perspectives for the future treatment of HUA.

## Graphical abstract

This review summarizes the characteristics, prevalence, and treatment strategies of hyperuricemia, and subsequently examines its pathogenic mechanisms together with the potential roles and regulatory pathways of the gut microbiota in its alleviation. It seeks to address gaps in current knowledge of microbiota‐mediated uric acid homeostasis and to provide new perspectives for future therapeutic approaches to hyperuricemia.

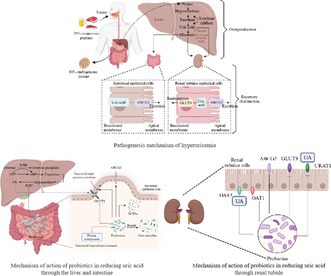

## 1 Introduction

Hyperuricemia (HUA) is a common metabolic disorder characterized by abnormally high concentrations of uric acid (UA) in the blood (Galozzi et al. 2021). The prevalence of HUA has shown an increasing trend worldwide over the past decades (Dong et al. 2025). According to WHO data, 20%–30% of adults worldwide suffer from HUA to varying degrees, which poses a significant public health burden. This condition often arises from an imbalance in UA metabolism, leading to excessive production and impaired excretion. HUA can result in immediate symptoms, such as gouty arthritis, and increase the risk of kidney stones, chronic kidney disease, and cardiovascular disease. Effective management of HUA is crucial to prevent complications such as gout attacks, nephrolithiasis, and chronic kidney disease, and maintain overall health (Dong et al. 2025; Du, Zong, et al. 2024). Currently, the primary treatments for HUA involve drug therapy and dietary changes; however, some medications used in treatment have been associated with risks such as liver and kidney toxicity, as well as other adverse events (Bardin and Richette 2017; Nielsen et al. 2018). Representative drugs such as allopurinol and febuxostat may cause serious adverse skin reactions, including diseases such as Stevens‐Johnson syndrome (SJS) and toxic epidermal necrolysis (TEN). Therefore, further investigation into the underlying mechanisms of HUA and the search for more natural, effective, and safer therapeutic options has become an urgent priority.

The gut microbiota is now recognized as a functional “vital organ” owing to its multidimensional connectivity with other body systems. This interconnectedness, referred to as the gut microbiota axis, underlies critical interactions between the host and its microbes via neural, endocrine, humoral, immune, and metabolic signaling pathways (Afzaal et al. 2022). Most human gut microorganisms are harmless and maintain mutually beneficial relationships with the host, contributing significantly to immune defense against pathogens (Bai et al. 2024; Li et al. 2022). The gut microbiota has been linked to numerous health conditions, including modulating immunity, mental disorders (like anxiety and depression), cardiovascular and metabolic diseases (such as hypertension, obesity, diabetes, and phlegm‐dampness constitution), as well as inflammatory bowel diseases and cancer (Chen, Wang, et al. 2023; Li et al. 2025; Yan et al. 2025; Zeng et al. 2024; Zhu et al. 2022). In metabolic diseases such as type 2 diabetes, obesity, and non‐alcoholic fatty liver disease, natural compounds and microbiota‐targeted therapies have demonstrated unique value (Li et al. 2021). Probiotics, prebiotics, and dietary interventions optimize the composition of gut microbiota, promote the production of short‐chain fatty acids (SCFAs), enhance intestinal barrier function, and reduce endotoxin release (Li et al. 2021). These mechanisms collectively regulate metabolic processes and alleviate inflammation, thus offering novel pathways for the prevention and treatment of metabolic diseases.

In recent years, research has indicated that patients with HUA often exhibit gut microbiome dysbiosis, which is commonly defined as a decrease in microbial diversity, an absence of beneficial microbes, or the presence of potentially harmful microorganisms, and that intestinal microorganisms play a significant role in the pathogenesis of HUA (Cao et al. 2023; Kasahara et al. 2023; Winter and Baumler 2023; Xie et al. 2022). The limitations of traditional therapeutic approaches have prompted researchers to turn their attention to emerging areas, among which the interaction between the gut microbiota and host metabolism has become a focus of research in recent years. Increasing evidence suggests that the gut microbiota plays a key role in the development of various metabolic diseases, providing a new perspective for exploring the pathogenesis and developing therapeutic strategies for HUA. Approximately 25% of UA is excreted into the intestine, where it is metabolized by the gut microbiome (e.g., *Lactobacillus*, *Bifidobacterium*) (Chu et al. 2021; Yin et al. 2022). These intestinal microorganisms can indirectly influence UA solubility and excretion efficiency by regulating the intestinal environment (e.g., pH and redox potential) or by acting synergistically with other flora. As a result, many studies are now focusing on the intestinal tract as a critical target for reducing UA levels by regulating the metabolism of the gut microbiome (Meng et al. 2023; Yamada et al. 2017).

In this paper, we describe the pathogenesis of HUA and the mechanisms through which the gut microbiome plays a therapeutic role, on the basis of an extensive review of the related literature. It provides a theoretical foundation for further research in this area and offers a new perspective on the future treatment of HUA.

## 2 Characteristics and Prevailing Conditions of HUA

HUA is a common chronic metabolic disorder that is associated with increased production and/or decreased excretion of UA. UA is a metabolic byproduct in the human body. Under normal conditions, the body effectively excretes UA through the kidneys and intestinal pathways. The latter accounts for around a third of total UA excretion, and maintaining UA concentrations within a reasonable range (Crawley et al. 2022; Jin et al. 2012) (Male: 208–428 μmol/L, Female: 149–357 μmol/L). In recent years, the incidence of HUA has increased rapidly because of changes in people's dietary habits and a reduction in physical labor. However, factors such as dietary imbalances, reduced kidney function, and genetic influences can lead to an imbalance in UA production or elimination. This imbalance can result in abnormally high levels of UA in the blood, known as HUA (Fasting serum UA levels > 420 μmol/L (7.0 mg/dL) on two different days). The complex interplay between UA production and excretion is a crucial factor in the pathogenesis of HUA. The development of HUA is frequently accompanied by the presence of concomitant pathological conditions (Yang et al. 2022, 2017). Prolonged elevated blood UA levels have been demonstrated to result in the deposition of urate in joints, ankles, and other distal joints of the limbs (Wrigley et al. 2020; Yang et al. 2017; Zhen and Gui 2017). The global emergence of HUA as a significant health concern has been well documented.

### 2.1 Characteristics of HUA

HUA is a metabolic disorder characterized by abnormally high blood UA levels. The WHO defines HUA as having fasting serum UA levels exceeding 420 μmol/L (7.0 mg/dL) twice on different days (Wang et al. 2020). The deposition of sodium urate crystals caused by excess UA is the main pathomechanism of gout disease, potentially leading to joint deformity, stiffness, and even kidney damage or uremia (Shan et al. 2021). Over 80% of the UA in the body is produced from endogenous purine metabolism, primarily from nucleic acids such as adenine and guanine released by damaged or dead cells. The remaining 20% comes from exogenous purines synthesized in the liver and intestine (Jung et al. 2020).

**FIGURE 1:**
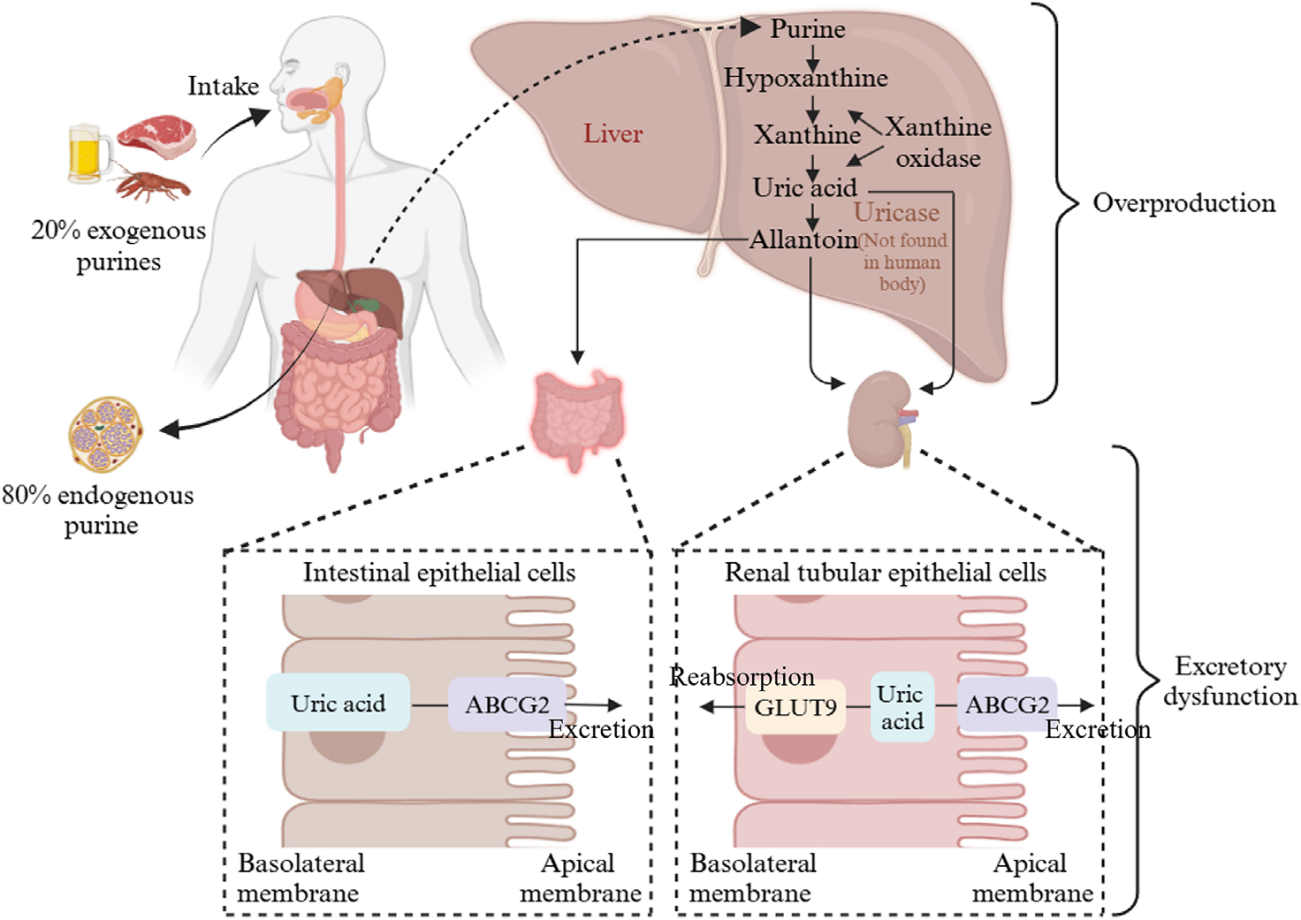
Pathogenesis mechanism of hyperuricemia.

Most mammals (such as dogs, cats, and pigs) possess uricase, which helps maintain their low baseline UA levels. Evolutionarily, humans lack uricase, the enzyme responsible for breaking down UA; this evolutionary loss exacerbates UA accumulation. As a result, UA cannot be metabolized into soluble and easily cleared allantoins. The kidney serves as the primary route for UA excretion, with two‐thirds being excreted via the renal pathway and one‐third through the intestine (Esche et al. 2020). Impaired glomerular filtration, enhanced tubular reabsorption, suppressed tubular secretion, and increased urate crystal deposition can all lead to reduced UA excretion, contributing to HUA. The related pathogenesis is summarized in Figure 1 (Esche et al. 2020; Jung et al. 2020; Lu et al. 2019). Furthermore, when UA crystals are recognized by the immune system as a foreign body, they activate neutrophils, macrophages, and other immune cells, prompting them to release pro‐inflammatory cytokines, such as interleukin‐1 beta (IL‐1β) and tumor necrosis factor‐alpha (TNF‐alpha), which triggers a strong inflammatory response, resulting in local redness, swelling, and pain; symptoms, and persistent inflammation and abnormal immune response may further aggravate tissue damage and promote disease progression (Li, Yuan, et al. 2023; Wilson and Saseen 2016). The persistent inflammation and abnormal immune response may further aggravate tissue damage and promote disease progression (Li, Yuan, et al. 2023).

### 2.2 The Current Status of Treatment of HUA

HUA is a potential predisposing factor for a wide range of severe health conditions, including cardiovascular disease, metabolic syndrome, hypertension, diabetes mellitus, and chronic kidney disease (Vareldzis et al. 2024; Yanai et al. 2021). In addition to these cardiometabolic disorders, HUA is considered a definite predisposing factor for the development of gout and urolithiasis (kidney stones) (Ichida et al. 2012). Emerging evidence also suggests that HUA may be potentially associated with osteoarthritis, contributing to joint inflammation and cartilage damage (Hisatome et al. 2018; Zhu et al. 2020). Furthermore, HUA has been linked to the impairment of vascular endothelial cell function, which can lead to compromised blood flow and cardiovascular complications (Zhen and Gui 2017). The far‐reaching negative health impacts of HUA highlight the importance of careful management and monitoring of UA levels. Elevated UA levels associated with HUA can have systemic effects, predisposing individuals to various severe cardiometabolic, musculoskeletal, and vascular disorders. Proactive interventions to address HUA are crucial to mitigate these substantial health risks.

The causes of HUA can be categorized into three main types: excessive UA production, inadequate UA excretion, and a combination of both (Lima et al. 2015). The concentration of UA in the blood is determined by the balance between the purine content of the diet and the body's synthesis and excretion of UA. However, an excessive increase in UA synthesis or insufficient UA excretion can lead to elevated serum UA levels, resulting in HUA (Giordano et al. 2015). Factors contributing to increased UA production or decreased UA excretion include obesity, genetic factors, deficiencies in key enzymes involved in purine metabolism, such as phosphoribosyl pyrophosphate (PRPP) synthetase and adenosine deaminase (ADA), enhanced purine oxidase activity, and abnormal expression of urate transporter proteins such as URAT1, GLUT9, and ABCG2 (Hamada et al. 2008; Han et al. 2018; Kolz et al. 2009; Li et al. 2020; Maiuolo et al. 2023; Yano et al. 2014).

Current therapeutic approaches for lowering UA levels can be broadly categorized into two strategies: reducing UA production and promoting UA excretion. Reduced UA production can be achieved with xanthine oxidase (XOD) inhibitors such as allopurinol or febuxostat, which block the conversion of purine precursors to UA. Dietary modifications, such as limiting the intake of purine‐rich foods (animal livers, some seafood, and so on), can also help lower UA levels. Methods to enhance UA excretion include supplementation with UA oxidase to convert UA into more soluble allantoin and the use of drugs such as probenecid, benzbromarone, and sulfinpyrazone to stimulate renal UA transporters and promote urinary UA excretion (Martens et al. 2020; Matsuo et al. 2020).

However, these therapeutic approaches have limitations and potential adverse effects. A randomized, double‐blind, non‐inferiority trial enrolled 940 patients with gout, who received either allopurinol or febuxostat in combination with anti‐inflammatory prophylaxis. The results showed that both medications demonstrated similar efficacy in controlling gout flares, lowering serum UA levels, and ensuring overall safety (O'Dell et al. 2022). Singh et al. conducted a propensity‐matched analysis using U.S. Medicare data involving over 23,000 elderly gout patients and found that febuxostat was associated with a significantly higher risk of atrial fibrillation (HR = 1.25), especially at the 80 mg dose during the first 6 months of treatment (Singh and Cleveland 2019). Kang et al. (2021) performed a propensity score–matched cohort study including 124,434 newly diagnosed gout patients and reported a higher incidence of cardiovascular events and all‐cause mortality with allopurinol compared to benzbromarone (HR = 1.22 and 1.66, respectively) (Kang et al. 2021). Moreover, benzbromarone may pose hepatotoxicity risks (Dalbeth et al. 2019). Although these pharmacological strategies are effective in reducing UA, their long‐term safety and consistency across populations remain uncertain, often constrained by limited mechanistic understanding and a lack of standardized treatment protocols. Therefore, there is a need to explore novel, non‐toxic, and efficient approaches for managing HUA.

Recent research has highlighted the gut microbiome as a potential target for managing HUA. Alterations in the composition and function of gut microbiota have been shown to influence serum UA levels and metabolic pathways relevant to HUA. Recent advances suggest that modulation of the gut microbiome could represent a promising new therapeutic strategy, warranting further investigation into the underlying mechanisms and clinical applications.

## 3 Mechanism of UA Action In Vivo

**FIGURE 2:**
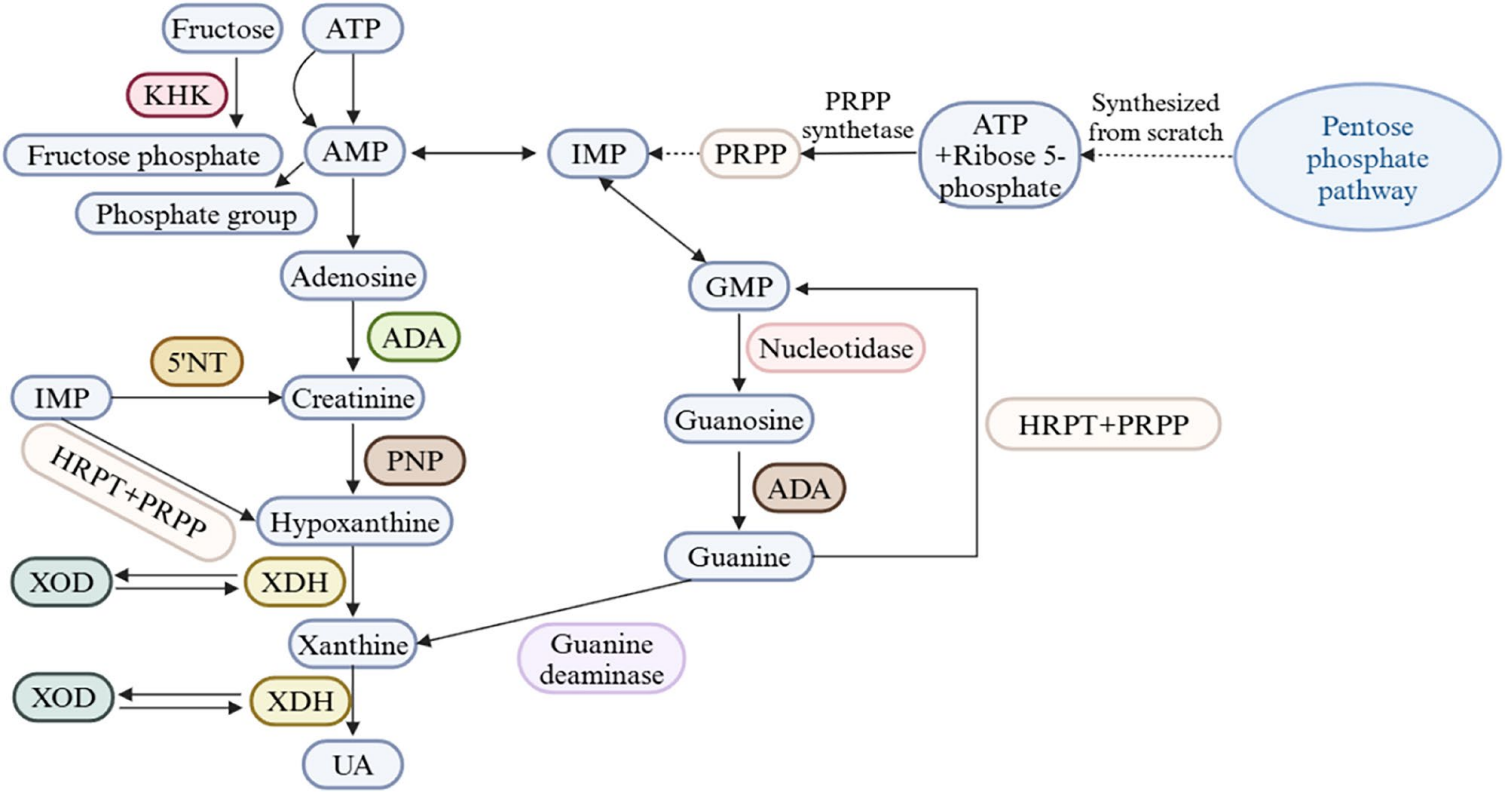
Production pathways of UA in the body.

UA is the terminal metabolite of purine degradation in humans. Approximately 80% of UA is produced through the metabolism of purines within the body, whereas the remaining 20% comes from the consumption of purine‐rich foods (Pan et al. 2020). Additionally, some studies have indicated that hepatic amino acids can also contribute to purine production via the de novo synthesis pathway, thereby promoting UA generation. UA is primarily produced in the liver, intestines, muscles, endothelium, and kidneys, and is excreted through the kidneys and intestines (Jalal et al. 2013). Figure 2 illustrates the mechanism of UA production in the human body. The process starts with ribose 5‐phosphate and ATP combining via PRPP synthetase to form PRPP. PRPP undergoes reactions to generate inosine monophosphate (IMP), which interconverts with adenosine monophosphate (AMP) and guanosine monophosphate (GMP) (Ames et al. 1981; Stewart et al. 2019; Waring 2002). AMP is converted to IMP and adenosine by 5′‐nucleotidase (5′NT); adenosine becomes inosine via ADA (Waring et al. 2001). GMP is converted to guanosine by nucleotidase and purine nucleotide phosphorylase (Frei et al. 1989; Simic and Jovanovic 1989).

Conversely, it has been demonstrated that UA is an antioxidant only in hydrophilic environments and may function as a pro‐inflammatory factor involved in the generation of intracellular oxidants through nicotinamide adenine dinucleotide oxidase‐dependent pathways. This process potentially leads to oxidative stress and induces dysfunction in mitochondria, the endothelium, proximal renal tubules, and other tissues (Choi et al. 2014; Sautin and Johnson 2008; Xiao et al. 2015). Furthermore, UA may be linked to altered nitric oxide release in the endothelium and changes in acetylcholine‐induced vasodilation (Waring et al. 2000). When UA levels exceed the physiological range, as seen in HUA, the body's antioxidant capacity becomes overwhelmed. A recent high‐impact review points out that high UA can mediate the innate immune response. It activates the NLRP3 inflammasome, leading to the maturation and release of interleukin‐1β (IL‐1β), which in turn triggers a robust inflammatory reaction. Moreover, in the process of XOR‐mediated UA production, a large amount of reactive oxygen species (ROS) is generated. Excessive ROS can react with UA, further promoting oxidative stress and inflammation (Du, Zong, et al. 2024).

The roles of UA as both an oxidant and an antioxidant are not fully understood and remain under investigation. However, it is well documented that persistently elevated UA levels above the physiological range are associated with the development of several conditions, such as gout, urinary calculi, and kidney stones (Frei et al. 1989; Yamanaka 2011). Overall, UA exhibits a dual nature, exerting both protective and harmful effects within the body. Further studies are needed to clarify the mechanisms underlying these effects, including studies on microbiome‐mediated modulation of UA's redox activity and to determine the physiological balance between its oxidative and antioxidant activities.

There are close interactions between the UA metabolic pathway and the enzymes and metabolites of intestinal microorganisms (Wang et al. 2022). Enzymes produced by intestinal microorganisms (e.g., enzymes involved in purine metabolism) can directly affect the catabolism of UA precursor substances, whereas their metabolites, SCFAs, indirectly contribute to the metabolism of UA by modulating the function of the intestinal barrier and the state of systemic inflammation (Wang and Ye 2024). In addition, xanthine oxidase inhibitors of microbial origin can inhibit the activity of key enzymes of UA production and thus regulate the metabolic homeostasis of the UA pathway (Rao et al. 2024), and these interactions provide new perspectives for understanding the mechanism of UA metabolic disorder.

## 4 Pathogenic Mechanisms of HUA

Genetic and dietary factors significantly influence HUA. Genetic factors play an important role in the development of HUA, and if there is a family history of the condition, the risk of developing it increases. Dietary habits are also an important cause of HUA. Excessive intake of purine‐rich foods (e.g., visceral foods, seafood, and red meat), sugary drinks, and alcohol promotes UA production and interferes with UA excretion, resulting in elevated blood UA levels.

Genetic factors lay the foundational framework of gut microbiota composition through gene polymorphisms (such as ABCG2 gene variants), whereas dietary components like high‐purine and high‐fat diets dynamically reshape microbial structure (e.g., increasing the abundance of urate‐producing bacteria). These two factors collectively influence UA metabolism by regulating microbial metabolic activity, impacting UA production and excretion. Meanwhile, genetically determined activities of UA‐metabolizing enzymes (e.g., xanthine oxidase) synergize with diet‐induced microbial metabolites (e.g., SCFAs and phenols) to alter key enzymatic activities in UA metabolic pathways and amplify disease risks via the inflammation–oxidative stress network, making genetically susceptible individuals more prone to gout, metabolic syndrome, and other diseases under specific dietary exposure.

### 4.1 Genetic Factors

Genetic predispositions play a key role in the development of HUA and gout. Early studies identified several rare single‐gene disorders, such as deficiencies in enzymes involved in purine metabolism and abnormalities in the renal transport of UA, which can lead to severe HUA and gout. Although these single‐gene disorders are uncommon, they provide important insights into the molecular mechanisms regulating UA metabolism. In recent years, large‐scale genome‐wide association studies (GWAS) have identified several common gene loci associated with serum UA levels and gout susceptibility. The most important of these include SLC2A9, ABCG2, and SLC22A12, which encode renal and intestinal UA transport proteins (Merriman and Dalbeth 2011). Variations in these genes regulate the uptake and secretion of UA in the kidneys, thereby affecting serum UA concentrations and consequently altering the risk of gout development in individuals. It has been found that variants in these UA‐related genes account for approximately 3%–5% of the variation in serum UA levels.

In addition to genes directly related to UA metabolism, a GWAS identified a few loci related to energy metabolism, inflammation, and other processes, which may also affect UA metabolism and gout development through indirect mechanisms. This suggests that UA metabolism is a complex process involving the coordinated regulation of multiple physiological systems (Tin et al. 2019). However, the variation in serum UA levels explained by these genetic factors is too small to be used alone for gout risk prediction. Furthermore, UA‐related genetic variants were not significantly associated with cardiovascular outcomes, suggesting that serum UA levels may not be an independent risk factor for cardiovascular disease (Martinez‐Quintana et al. 2016). In addition, specific HLA genes are associated with severe hydroxypurine reactivity, which can be used to screen for and prevent adverse drug reactions. This provides new ideas for the individualization of UA‐lowering therapies.

Overall, the findings from GWAS studies have improved our understanding of the genetic basis of UA metabolism and gout. However, further research is required to fully elucidate the complex mechanisms involved and to develop more effective and personalized treatment approaches. Kolz et al. (2009) conducted a meta‐analysis of 14 GWAS involving 28,141 individuals of European descent. The study identified 954 significantly associated SNPs across nine genetic loci, including five novel loci: SLC22A11, SLC16A9, GCKR, LRRC16A, and the region near PDZK1. Previously known UA–associated genes such as SLC2A9, ABCG2, SLC17A1, and SLC22A12 were also validated. These newly identified loci reveal novel biological pathways related to UA metabolism. Notably, the minor allele of SLC2A9 rs734553 was more strongly associated with UA reduction in females, whereas ABCG2 rs2231142 significantly increased serum UA levels in males. Additionally, the SLC16A9 rs12356193 variant was strongly correlated with the levels of DL‐carnitine and propionylcarnitine—two metabolites closely associated with serum UA—suggesting a complex role in urate regulation. Overall, this study not only expands our understanding of the genetic regulation of UA metabolism but also highlights solute transporters as promising therapeutic targets for HUA and gout, providing new directions for future mechanistic studies and drug development. Using a GWAS, Tin et al. (Köttgen et al. 2013) identified 18 new genetic loci associated with serum UA concentrations in European and Asian populations. These loci are involved in various biological processes, such as UA transport, renal function, and inflammation regulation, providing important insights into the genetic basis of UA metabolism regulation. Vitart et al. (Li et al. 2007) found that the UA transporter protein encoded by SLC2A9 plays a key role in UA metabolism and that polymorphisms in SLC2A9 can significantly affect serum UA levels, thereby increasing the risk of developing HUA and gout. Köttgen et al. (Major et al. 2018) identified 18 new loci associated with serum UA levels through a large‐scale genomic association analysis, providing important clues for exploring the role of genetic factors in the pathogenesis of HUA. Yerlikaya et al. (2017) demonstrated that GLUT9 is an important genetic factor in the regulation of serum UA levels and that polymorphisms in GLUT9 are significantly associated with the development of HUA.

Genetic factors play an important role in the pathogenesis of HUA and gout, and GWAS have revealed several key genes that regulate UA metabolism, providing new insights into the pathogenesis of this disease. However, the clinical application of GWAS must be further explored because of large individual differences. The future of precision medicine requires a comprehensive assessment and intervention that takes both genetic and environmental factors into account. Building on this foundation, future efforts may explore personalized risk assessment on the basis of genetic susceptibility to optimize the management of HUA. In addition, targeted interventions aimed at regulating specific functional gene variants could offer more precise and sustained therapeutic options for high‐risk individuals. The advancement of these strategies is expected to facilitate a shift in HUA treatment from traditional symptom‐based approaches to mechanism‐driven, personalized precision interventions.

### 4.2 Dietary Factors

In recent years, driven by changes in dietary habits and lifestyle, the incidence of HUA has been steadily increasing (Cao et al. 2017). Studies have shown that diets high in fructose, purines (such as those found in animal offal, red meat, and seafood), and fats can directly cause a marked increase in the levels of UA, urea nitrogen, and creatinine, as well as the production of inflammation in the body. These dietary factors can also directly alter the composition of intestinal microorganisms, such as decreasing the proportion of probiotic bacteria like *Lactobacillus* and *Bifidobacterium*, whereas significantly increasing the proportion of pathogenic bacteria. This shift leads to the accumulation of UA, reduced excretion, and increased XOD activity, thereby exacerbating HUA symptoms, thus exacerbating the symptoms of HUA. Fructose intake elevates xanthine oxidase (XOD) activity through hepatic fructokinase‐mediated ATP depletion, which accelerates purine degradation and converts xanthine dehydrogenase to XOD, thereby enhancing UA production and reactive oxygen species (ROS) generation. Concomitantly, fructose‐induced ROS and UA trigger inflammasome activation (e.g., NLRP3) and NF‐κB signaling, promoting the release of pro‐inflammatory cytokines (TNF‐α, IL‐6) and exacerbating systemic low‐grade inflammation via gut‐liver axis disruption or direct hepatic stress responses. Additionally, HUA can worsen gut microbiome disorders and UA levels, creating a vicious cycle (Massy and Drueke 2021; Zmora et al. 2019). UA accumulation exacerbates intestinal dysbiosis by inducing gut inflammation, disrupting intestinal barrier integrity, or altering gut pH, thereby promoting the overgrowth of pro‐inflammatory microbiota and inhibiting the metabolic activity of beneficial bacteria. This imbalanced state further drives the progression of metabolic disorders like insulin resistance, lipid metabolism dysfunction, and obesity by reducing the production of protective metabolites (e.g., SCFAs), triggering endotoxemia, or activating systemic oxidative stress pathways (Massy and Drueke 2021; Zmora et al. 2019).

**FIGURE 3:**
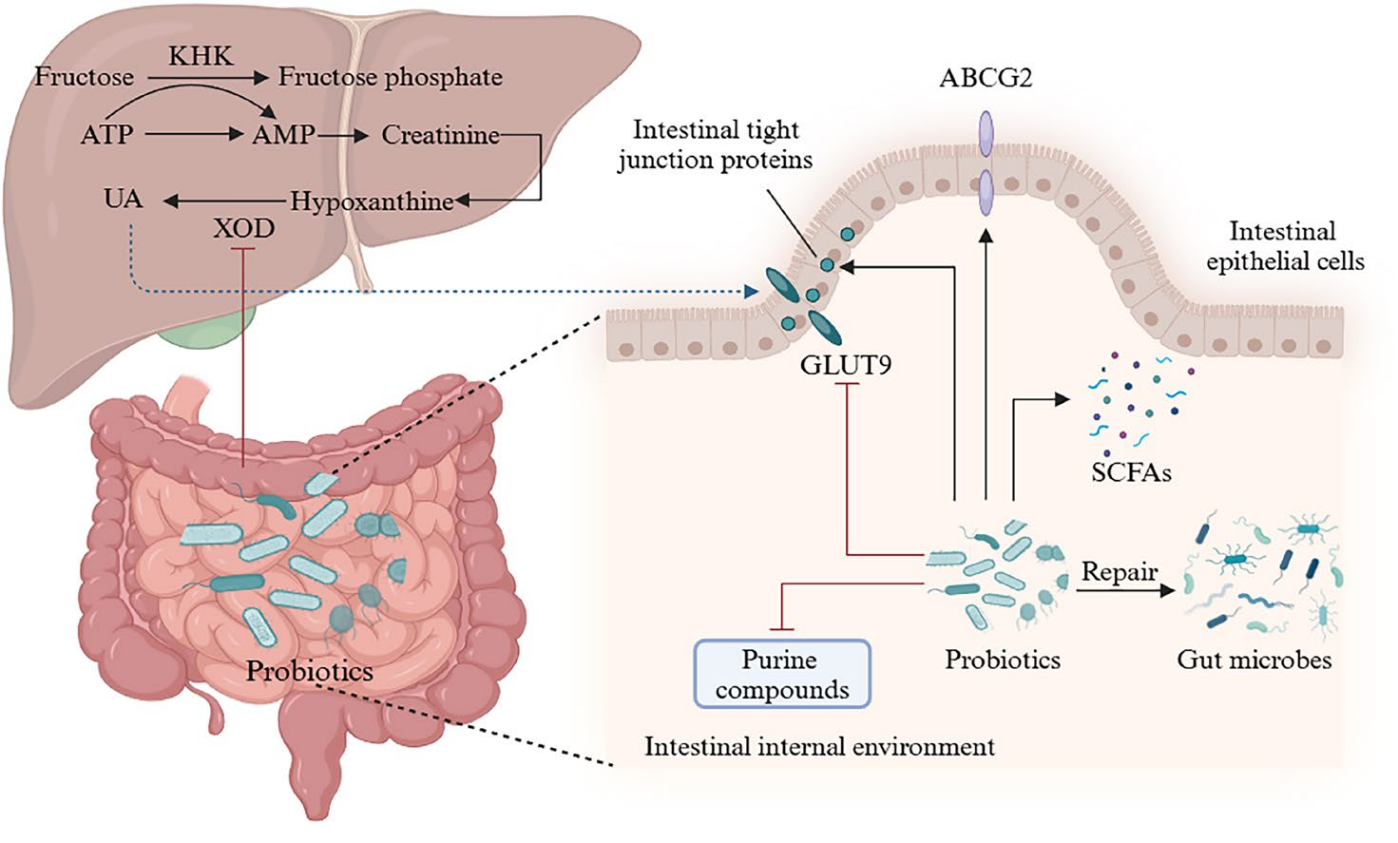
Mechanism of action of probiotics in reducing UA through the liver and intestine.

Diet is a key factor influencing the structure and function of gastrointestinal microorganisms, which can alter their diversity and result in changes to microbial metabolites (Nakagawa et al. 2019; Vedder et al. 2019). This effect worsens over time. Therefore, avoiding long‐term intake of HUA‐inducing foods, such as sweets, seafood, and fried foods (Figure 3), represents a feasible strategy to prevent elevated UA levels and intestinal dysfunction. Specific foods, such as dairy products, cherries, and celery, can help relieve HUA symptoms (Chen, Luo, et al. 2023; Luyun et al. 2018). Furthermore, dietary recommendations for managing HUA should be developed within a broader public health framework, especially in the context of the rising global burden of metabolic disorders. Policy guidance, nutrition education, and environmental interventions should be employed to promote healthy eating patterns across the population, thereby reducing the incidence of HUA and related metabolic disturbances.

#### 4.2.1 High‐Sugar Diet

Fructose, the sweetest natural sugar, is widely used in foods such as soft drinks, fruit juices, and baked goods. When fructose is absorbed by the small intestine and metabolized in the liver, a large amount of ATP is consumed during catabolism. This process depletes ATP and produces excess AMP, which then degrades into hypoxanthine (Yamada and Sherman 1981). Hypoxanthine is further broken down into metabolic intermediates such as UA and lactic acid, leading to abnormally high blood UA levels (Figure 2) (Park et al. 2017; Zhang, Li, et al. 2020). The catabolism of fructose also induces metabolic stress, inflammatory responses, and endothelial dysfunction, which can contribute to the development of metabolic diseases, including HUA, diabetes mellitus, obesity, and atherosclerosis (Ebrahimpour‐Koujan et al. 2020).

In recent years, fructose intake has increased annually, and there is a positive correlation between fructose consumption and the incidence of HUA in the population (Do et al. 2018; Silva et al. 2018; Zhang, Bian, et al. 2020). Massy and Drueke (2021) found that a high‐fructose diet can significantly reduce intestinal microbial diversity in a short time, increasing the abundance of *Firmicutes* and decreasing the abundance of *Mycobacterium avium* , which further decreases the content of SCFAs in the gut. Additionally, a high‐fructose diet induces an “endotoxemic state” in the host, leading to chronic inflammation. Similarly, Do et al. (Sun et al. 2010) found that in mice fed a high‐fructose diet, gut microbial diversity was reduced, with a lower proportion of *Mycobacterium* species and a significantly higher proportion of *Aspergillus* species. Silva et al. (Yu et al. 2013) showed that fructose‐fed groups had increased production of bile acids and taurine compared to normal mice, which induced metabolic disturbances in the host and further compromised the integrity of the intestinal barrier. In conclusion, managing fructose intake may help restore gut microbiome balance and reduce HUA incidence. However, some studies have found no significant relationship between a high‐fructose diet and the incidence of HUA. Global consumption of high‐fructose diets exhibits significant regional variations: North America is dominated by high‐fructose corn syrup with large consumption volumes; Europe, constrained by policies, favors sucrose; Asia sees rapid growth with distinct local characteristics; and the Middle East features a coexistence of traditional high‐fructose foods and industrial beverages. These differences are closely linked to economic development, food industries, policies, and cultural practices. For example, Sun et al. (2020) analyzed data from the American Health and Nutrition Examination Survey (AHANES) database from 1999 to 2004 and found no correlation between a high‐fructose diet and the prevalence of HUA in healthy populations. Yu et al. (2018) also found that the metabolic characteristics of high‐fructose corn syrup and sucrose intake were similar, with a lower urinary tract temperature than that of high‐fructose corn syrup. Furthermore, the metabolic characteristics of high‐fructose corn syrup and sucrose intake in healthy humans were similar, and UA levels did not significantly differ. An acute fructose challenge study found that individuals carrying specific SNPs experienced a more significant increase in serum uric acid levels after consuming a high‐fructose beverage, indicating that genetic polymorphisms play a role in short‐term urate metabolism (Zhang, Mass, et al. 2022). Therefore, the relationship between fructose and HUA may be influenced by differences in research samples and the diversity of fructose sources. The “individualized” interference of population characteristics, manifesting in differences in health baselines, regional lifestyle patterns, and genetic backgrounds, leads to divergent UA metabolic responses to fructose across populations. In terms of fructose source diversity, natural fructose—coexisting with components like dietary fiber—exerts weaker metabolic impacts, whereas processed fructose, because of its high dosage and synergistic effects with other ingredients, more readily elevates UA levels. Therefore, further research is needed to determine whether a high‐fructose diet is likely to induce HUA.

#### 4.2.2 High‐Purine Diet

Purines are natural substances found in nearly all foods, and their content and type significantly influence UA levels. The intake of foods with high purine content, such as seafood, meat, and alcoholic beverages, increases the nucleic acids in the body, leading to excessive levels of the metabolite UA, which contributes to the development of HUA (Liu et al. 2020). This can also alter the structure and diversity of the gut microbiome. Huang et al. (Sheng‐Nan et al. 2015) found that feeding quail high‐purine feed made by mixing ordinary feed with 15 g/kg dried yeast powder induced HUA and changed the structure of the quail's gut microbiome. They also discovered that the contents of intestinal flora metabolites, such as lipopolysaccharides (LPSs) and XOD, were positively correlated with blood UA levels. Therefore, the intake of high‐purine foods can contribute to the development of HUA not only by increasing UA levels but also by modifying the gut microbiome composition.

Cao et al. (2017) found that in a mouse model of diet‐induced HUA, excessive nucleic acid intake caused elevated UA levels, which subsequently altered the structure and diversity of the gut microbiome. Similarly, Liu et al. (2020) established a rat model of high‐purine diet‐induced HUA and used 16S rDNA sequencing to analyze changes in intestinal microorganisms, identifying *Vallitalea*, *Christensenella*, and *Insolitispirillum* as being correlated with HUA. To further explore the role of intestinal bacteria in high‐purine‐induced HUA, the intestinal microorganisms *Bifidobacterium* and *Lactobacillus* in mice were analyzed through 16S rDNA sequencing. Additionally, fecal microbiota transplants from hyperuricemic rats to normal rats were performed to compare UA levels. The results revealed that fecal microbiota from hyperuricemic rats caused a significant increase in UA levels in recipient rats, indicating that the gut microbiota plays an important role in HUA induced by a high‐purine diet. Moreover, individual genetic differences can influence serum uric acid levels following purine intake. A large‐scale prospective study on the basis of the Korean KoGES‐HEXA cohort (44,053 participants) systematically evaluated the association between red and processed meat consumption and the risk of developing HUA. The results showed that among individuals with high genetic risk, higher intake of red and processed meat was associated with a significantly increased risk of HUA, with relative risks of 2.72 for men and 3.28 for women. These findings suggest a significant interaction between dietary factors and genetic susceptibility.

#### 4.2.3 High‐Fat Diet

Long‐term intake of high‐fat foods can increase UA levels and induce HUA. Yu et al. (2018) found that feeding rats a high‐fat diet containing 10% yeast extract for 6 weeks successfully induced HUA, leading to changes in the gut microbiome. The changes observed in *Anabaena* and *Bifidobacterium* species were consistent with findings by Guo et al. (2016). Additionally, Hsu et al. (2019) showed that rats on high‐fat diets had significantly higher body weights and UA levels compared to those on regular diets, with *Lactobacillus plantarum* GKM3 potentially reducing UA levels by altering the intestinal microflora composition. Sun et al. (2020) observed that C57BL/6 mice fed a long‐term high‐fat diet developed proteinuria, increased blood urea nitrogen and creatinine, kidney dysfunction, and increased tubular cell apoptosis. These findings indicate that a high‐fructose, high‐creatinine, and high‐fat diet is associated with proteinuria, creatinine accumulation, renal dysfunction, and increased apoptosis of renal tubular cells. Therefore, limiting high‐fat diet intake can help prevent HUA. A high‐fat diet can induce obesity, trigger insulin resistance, and impair renal UA excretion. Meanwhile, fatty acids and inflammatory cytokines secreted by adipocytes activate hepatic xanthine oxidase, promoting UA synthesis. Recent studies indicate that saturated fats may indirectly elevate UA by exacerbating metabolic disorders and inflammatory responses, whereas unsaturated fats might exert positive effects on UA regulation by improving the metabolic microenvironment—though specific mechanisms require further validation.

## 5 Overview of Gut Microbiome

The intestinal flora consists of a diverse array of microorganisms that colonize the human gut and play an essential role in regulating energy and metabolism (Tran et al. 2015). Structural changes or imbalances in the gut microbiome can lead to metabolic disorders (Ding et al. 2022; Li, Li, et al. 2023; Shen et al. 2024). The use of the intestinal microbiome as an entry point to explore the pathogenesis of diseases has become a new area of focus, particularly for metabolism‐related conditions, such as easing the symptoms of irritable bowel syndrome and HUA (Angelucci et al. 2019; Bian et al. 2020; Liu et al. 2020; Xie et al. 2023).

According to the gut–renal axis theory, dysbiosis damages the intestinal barrier, leading to increased intestinal permeability. This allows various toxins to enter the bloodstream and reach the kidneys, potentially triggering inflammation (Nakai et al. 2006). Recent studies have found that this inflammation can affect the expression of UA transporter proteins, which may lead to increased serum UA levels. Additionally, changes in the gut microbiome have been shown to directly influence UA metabolism. An extensive literature review has revealed that modulating the structure of the gut microbiome may have a therapeutic effect on HUA (Nomura et al. 2013).

## 6 The Relationship Between Intestinal Microbes and HUA

HUA, a disorder in purine metabolism, is often associated with elevated levels of pro‐inflammatory factors, oxidative stress, and dysregulation of the intestinal microbiome (Xu et al. 2019; Yu et al. 2018). Emerging research has demonstrated that the ecological imbalance of the gut microbiome is closely linked to HUA. The intestine plays a critical role in the excretion of UA, accounting for one‐third of its elimination (Shao et al. 2017). Additionally, resident gut microorganisms are essential in metabolizing the UA secreted into the intestine. The gut microbiome also produces metabolites, such as SCFAs, that can influence host metabolism and overall health. Long‐term consumption of high‐fructose, high‐purine, and high‐fat foods alters the structure and composition of the intestinal microbiome, which in turn affects the gut's role in purine and UA metabolism and excretion, leading to elevated blood UA levels (Xu et al. 2019). Conversely, elevated UA levels can induce chronic inflammation in the body and intestinal tract, changing the internal environment and further affecting the composition and abundance of the gut microbial community. This creates a bidirectional and mutually reinforcing relationship between the intestinal microbiome and HUA.

Interestingly, certain bacterial genera that increase in hyperuricemic animal models may correlate positively with higher blood UA levels. This suggests that targeted modulation of the gut microbiome may be a promising therapeutic strategy for managing HUA and related metabolic disorders. In patients with gout, Guo et al. (2016) observed an increased abundance of *Enterococcus faecalis* and *E. xylanolytica*, whereas *Bifidobacterium pusillus* and *B. pseudostreptococcus* were significantly reduced. In contrast, *Bifidobacterium pusillus* and *Bifidobacterium pseudostreptococcus* were found in lower abundance by 16S rDNA sequencing. Shao et al. (2017) analyzed 26 fecal samples from patients with gout using nuclear magnetic resonance hydrogen spectrometry and high‐throughput sequencing. They discovered that the diversity of fecal flora was significantly reduced in gout patients compared to healthy individuals, whereas pathogenic bacterial taxa such as *Mycobacterium* spp., *Zygomonas* spp., and *Anaerobic cordyceps*. were significantly more abundant. For more information on the characteristics of the gut microbiome in patients with HUA, refer to Table 1.

**TABLE 1:** Characteristics of intestinal microbiome in the hyperuricemia organism.

Study design	Contributing factor	Characterization of intestinal microorganisms	Causality (correlation vs. causation)	References
Human	Gout	*Mycobacterium* spp., *Zygomonas* spp., and *Anaerobic cordyceps* were significantly more abundant	Correlation	Shao et al. (2017)
Human	Gout	*Bacteroides caccae* and *Bacteroides xylanisolvens* are enriched, yet *Faecalibacterium prausnitzii* and *Bifidobacterium pseudocatenulatum* are depleted	Correlation	Guo et al. (2016)
Animal (wistar rat)	10% yeast extract for high‐fat feeds	There was a significant decrease in the genera *Prevotella*, *Extremophilic archaea*, and *Lactobacillus*; and an increase in the number of *Aspergillus* spp. that can secrete xanthine dehydrogenase to convert purines to uric acid	Correlation	Hsu et al. (2019)
Animal (wistar rats)	Yeast feeds and a high‐purine diet	*Vallitalea*, *Christensenella*, and *Insolitispirillum* were enriched	Causation (Fecal microbiota transplantation)	Liu et al. (2020)
Animal (c57BL/6 rat)	Uox‐KO mice	After co‐housing with Uox‐KO mice, the abundance of *Ileibacterium*, *Anaerotruncus*, and *Roseburia* significantly increased in the wild‐type mice	Causation (co‐housing experiment)	Song et al. (2022)
Animal (quails)	Yeast feed	There were changes in the abundance of *Mycobacterium anisopliae*, *Mycobacterium thickum*, and *Mycobacterium anisopliae* and inflammatory changes in the cecum tissue	Correlation	Agus et al. (2021)

In summary, the gut microbiome is related to the pathogenesis of HUA and may become a new target for the alleviation and treatment of HUA and related diseases in the future. With continuous advancements in research on the relationship between HUA and gut microbiome, its pathogenesis has been further elucidated, and the characterization of gut microbiome has emerged as a new target for treatment strategies. Armour et al. (2019) studied 2000 human fecal flora samples and found that changes in α‐diversity, β‐diversity, and β‐dispersity were specifically correlated with rheumatoid arthritis. They constructed a regression model on the basis of changes in microbial function, which accurately differentiated the disease from other conditions. This approach could offer a new diagnostic method for diagnosing patients with HUA or gout on the basis of differences in fecal microbiota composition. This emerging evidence underscores the therapeutic promise of microbiota‐targeted interventions, such as probiotics, dietary modulation, and fecal microbiota transplantation (FMT). This suggests that, in the future, microbiota‐based biomarkers may be developed as non‐invasive tools for early diagnosis or risk stratification of HUA and gout. Moreover, integrating gut microbiome profiling into personalized treatment strategies may open new avenues for precision medicine approaches targeting metabolic disorders.

## 7 Mechanisms of Action of Gut Microbiome in Alleviating HUA

The gut microbiome may alleviate HUA by restoring intestinal microbial diversity and directly acting on intestinal epithelial cells or producing metabolic byproducts to promote the expression of tight junction proteins (e.g., ZO‐1 and occludin). Elevated levels of tight junction proteins help inhibit the transmission of endotoxins and inflammatory factors within the intestinal tract, thereby reducing the inflammatory response (Liang et al. 2022; Singh et al. 2024). Relevant studies have shown that SCFAs produced by gut microbiome can inhibit XOD activity, SCFAs produced by gut microbiota primarily inhibit xanthine oxidase (XOD) activity through indirect mechanisms, including regulating the inflammation‐oxidative stress axis, reprogramming metabolic pathways, activating antioxidant networks, and synergistically reducing XOD‐activating substrates and pro‐inflammatory signals via microbiota‐host interactions, suggesting that modulating the microbiome to increase SCFA production could lower UA levels. Studies have also shown that probiotics can effectively reduce UA levels in patients with HUA by regulating the gut microbiome (Table 2).

**TABLE 2:** Studies on the mechanism of action of probiotics in alleviating hyperuricemia.

Mechanism	Probiotics	Action effect	References
Inhibit the activity of XOD	10^9^ CFU/mL *Lactobacillus plantarum* UA149	Decreased serum UA levels	Cao, Liu, et al. (2022)
	5 × 10^9^ CFU/mL *Lactobacillus rhamnosus* CCFM1130, CCFM1131, and *Lactobacillus royale* CCFM1132	The XOD activity and UA levels in the liver and serum of hyperuricemia mice were significantly decreased	Xu et al. (2022)
	3 × 10^9^ CFU/mL *Lactobacillus paracasei* MJM60396	Reduced XOD activity by 81%	Kilstrup et al. (2005)
	*Lactobacillus paracasei* X11 and *Lactobacillus plantarum* Q7	Decreased XOD and ADA levels	Lee et al. (2022), Ma et al. (2016)
Promote purine degradation or metabolism	*Lactobacillus gasseri* PA‐3	Through the uptake and degradation of inosine, guanosine, and adenosine in the gut as well as the uptake of IMP, GMP, and AMP	Stow and Bronk (1993), Yamada et al. (2018)
	10^9^ CFU/kg/day *Lactobacillus* DM9218	Degradation rate of nucleotides and nucleosides	Wang et al. (2019)
	*Lactobacillus shortcombicus* DM9218	Absorption of inosine and guanosine reduces UA	Buzard et al. (1952)
Repair the gut barrier and regulate the gut microbiota	10^8^ CFU *Lactobacillus fermentum* JL‐3	Improve the structure and function of gut microbiome and maintain a stable state of intestinal microbial community	Wu et al. (2021)
	*Lactobacillus paracasei* MJM60396	Increases the expression of ZO‐1 in intestinal epithelium and blocks and protects the intestinal barrier	Yamada et al. (2016)
	*Lactobacillus rhamnosus* GG produced soluble proteins p40 (0.1–1.0 μg/mL), and p75 (0.1–1.0 μg/mL)	Repair the damage caused by hydrogen peroxide to the barrier function and tight junction function of Caco‐2 cells	Seth et al. (2008)
	2 × 10^8^ CFU *Akkermansia muciniphila*	The expression of intestinal compact junction proteins claudin, occluding and ZO‐1 increased by 70.35%, 179.16% and 155.02%, respectively, which reduced intestinal permeability	Zhang, Liu, et al. (2022), Zhang, Mass, et al. (2022)
Accelerate UA excretion	*Lactobacillus paracasei* MJM60396	Increased the expression of kidney urate excretion transporters OAT1 mRNA and OAT3 mRNA, and decreased the expression of urate reabsorption proteins URAT1 mRNA and GLUT9 mRNA	Yamada et al. (2016)
	1 × 10^8^ CFU/mL *Lactobacillus rhamnosus* Fmb14	It inhibited the expression level of URAT1 in the kidney and promoted the expression of ABCG2 in the colon	Zhao, Jiang, et al. (2022), Zhao, Chen, et al. (2022)
	*Lactobacillus plantarum* Dad‐13, *Lactobacillus plantarum* Mut‐7, and *Lactobacillus casei* OL‐5	Up‐regulated ABCG2 gene expression and accelerated uric acid excretion	Handayani et al. (2018)

**FIGURE 4:**
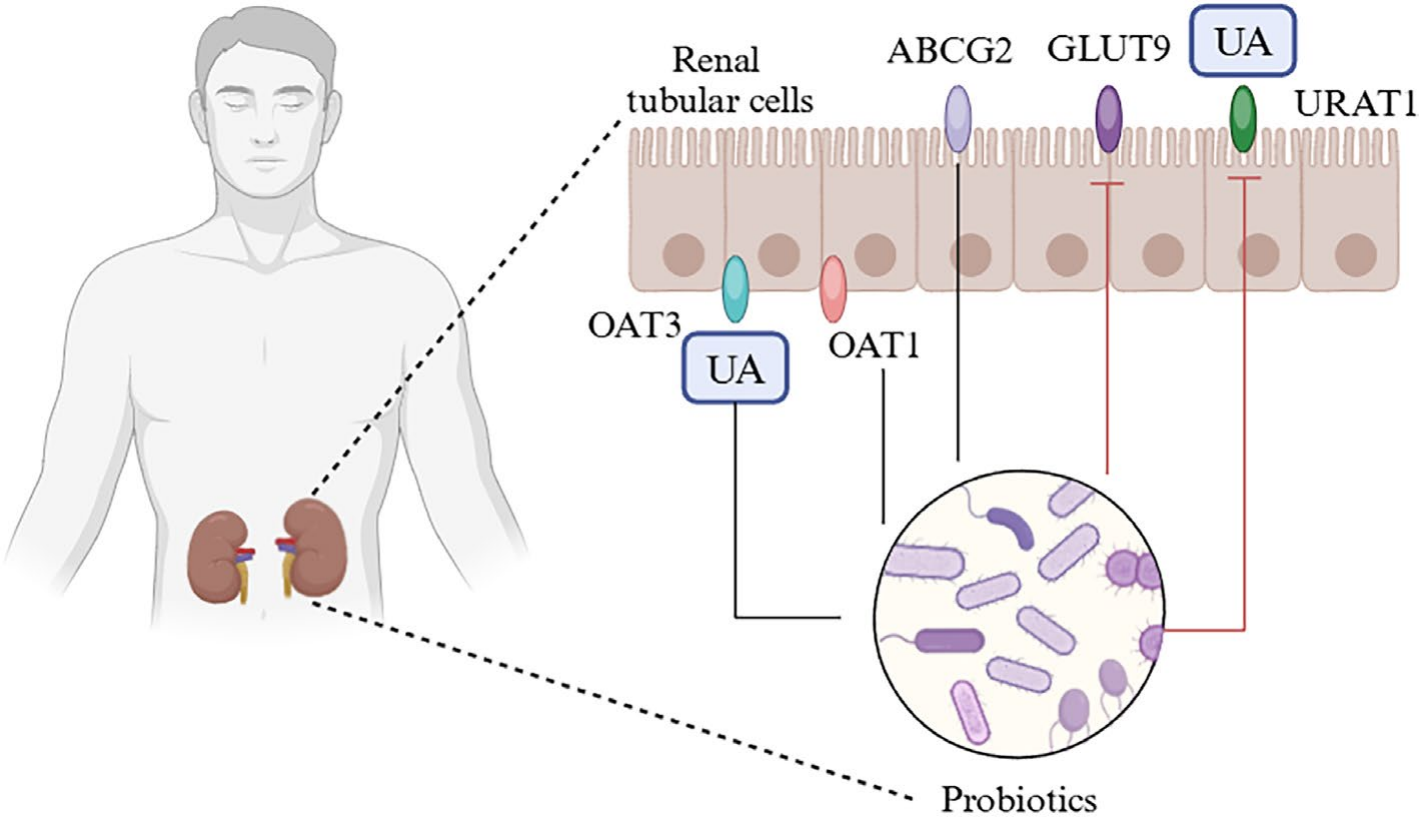
Mechanism of action of probiotics in reducing UA through the renal tubule.

Furthermore, the intestinal microbiome influences the renal excretion of UA. As shown in Figure 4, the gut microbiome can inhibit the expression of GLUT9 and URAT1 transporter proteins in renal proximal tubular cells, which are responsible for UA reabsorption (Arakawa et al. 2020). Conversely, the gut microbiome can promote the expression of transporters such as ABCG2, OAT1, and OAT3, which facilitate UA excretion (Zhao, Jiang, et al. 2022). This helps inhibit the reabsorption of urate by proximal renal tubular cells, promoting its elimination from the body. Additionally, the intestinal microbiome enhances UA excretion by promoting the expression of ABCG2 in the intestinal area and inhibiting the expression of GLUT9, further supporting its role in lowering UA levels (Mancikova et al. 2016).

### 7.1 Inhibition of XOD Activity

XOD is a key enzyme in UA synthesis, catalyzing the oxidation of hypoxanthine to xanthine, and then to UA. The intestinal microbiome plays an important role in alleviating HUA through its ability to inhibit UA production, leading to a reduction in UA levels. As illustrated in Figure 3, the proposed mechanism involves the gut microbiome inhibiting the activity of XOD, a key enzyme in the UA production pathway, thereby reducing UA synthesis. Furthermore, intestinal microbes may directly absorb, utilize, or degrade purine analogs, such as nucleotides, nucleosides, and purine bases within the gut, preventing their absorption by intestinal epithelial cells (Du, Wang, et al. 2024; Wei et al. 2022).

Inhibition of XOD activity is a proven method to reduce serum UA levels. Certain inflammatory factors or endotoxins, including LPSs and superoxide anions, can stimulate and increase XOD activity (Cao, Liu, et al. 2022; Ishii et al. 2014). Interestingly, some probiotic strains have been shown to inhibit UA production by improving intestinal barrier function and reducing LPS permeability, which in turn affects XOD expression and activity. For example, *L. plantarum* UA149, when administered to rats with HUA, significantly decreased XOD content and UA production, leading to lower serum UA levels. Similarly, *Lactobacillus rhamnosus* CCFM1130, CCFM1131, and *Lactobacillus royale* CCFM1132 were found to significantly reduce XOD activity and UA levels in the liver and serum of mice with HUA (Xu et al. 2022). Oral administration of *Bifidobacterium bifidum* also significantly inhibited the increase in XOD activity induced by UV irradiation in mice, possibly by reducing hydrogen peroxide production and oxidative damage (Cao, Bu, et al. 2022). Other probiotic strains, such as *Lactobacillus paracasei* X11 and *L. plantarum* Q7, have demonstrated the ability to decrease both XOD and ADA levels in mice (Lee et al. 2022; Ma et al. 2016). *L. paracasei* MJM60396 was shown to reduce XOD activity by 81% (Kilstrup et al. 2005). In a 2‐month randomized, double‐blind, placebo‐controlled clinical trial, 160 patients with gout received febuxostat in combination with either a daily dose of 3 × 10^10^ CFU of a multi‐strain probiotic powder (containing *Lactobacillus paracasei* Zhang, *L. plantarum* P‐8, *L. rhamnosus* Probio‐M9, *Bifidobacterium lactis* Probio‐M8, and *B. lactis* V9) or a placebo. The probiotic group showed a significant reduction in serum uric acid levels and a lower frequency of acute gout flares compared to the placebo group. However, only about half of the participants responded positively to probiotic intervention, as evidenced by reductions in serum UA, gout flare rate, and XOD levels. In contrast, non‐responders showed no significant difference from the placebo group, suggesting that individual responses may be closely linked to baseline gut microbiota composition (Zhao et al. 2024). Although some clinical studies have observed the adjunctive effects of probiotics in lowering serum uric acid levels among gout patients, their efficacy in individuals with hyperuricemia (HUA) remains insufficiently validated. Future large‐scale, long‐term prospective studies specifically targeting the HUA population are needed to clarify the clinical value of probiotic interventions.

Current studies focus on probiotics' ability to inhibit XOD activity and expression, as this can effectively reduce UA generation (Liu et al. 2024). Unlike drug therapy, probiotic interventions offer a gentle, side‐effect‐free treatment for HUA. The variability in probiotic efficacy and strain‐specific responses may lead to inconsistent individual reactions to the same strain, with the effectiveness of a specific strain potentially hindered by host factors like gut microbiota composition, metabolic status, and environmental influences, thus limiting universal applicability. Although the specific mechanisms by which probiotics inhibit XOD are still under investigation, their metabolites seem to play a key role. Further research in this area may lead to the development of novel probiotic‐based approaches for managing HUA and related metabolic disorders.

### 7.2 Degradation and Absorption of Purines in the Intestine

Purines in the human body exist mainly as purine nucleotides, purine nucleosides, and purine bases. Because of the absence of the uricase gene in humans, purine material is ultimately excreted from the body as UA. During purine metabolism, purine nucleotides are converted into purine nucleosides by nucleosidases, and purine nucleosides are converted into purine bases by purine nucleoside phosphorylase. These bases are then transformed into xanthines by XOD or ADA, and subsequently oxidized to UA by XOD (Cheng et al. 2022; Wei et al. 2022). Therefore, the degradation and uptake of purines by probiotics represents an important area of research for reducing UA levels. It has been demonstrated that some lactic acid bacteria can utilize purines, reducing intestinal absorption and, consequently, UA production (Yamada et al. 2016, 2017).

For instance, *L. paracasei* MJM60396 and *Lactobacillus formosanus* MJM60662 showed 100% degradation of inosine, guanosine, and adenosine in vitro, with the former also achieving 100% degradation of these compounds in lysates. *Lactobacillus gasseri* PA‐3 can reduce UA production by uptaking and degrading inosine, guanosine, and adenosine in the intestine, as well as by uptaking IMP, GMP, and AMP, thereby reducing the intestinal absorption of these and related purines in rats and exerting a UA‐lowering effect (Huang et al. 1993; Salati et al. 1984; Yamada et al. 2018). This strain has also been found to utilize nucleosides directly, which supports its UA‐lowering potential in the humans' intestinal tract (Kuo et al. 2021; Li et al. 2014). In a randomized, double‐blind, placebo‐controlled clinical trial, researchers evaluated the uric acid‐lowering effect of yogurt containing *Lactobacillus gasseri* PA‐3 in patients with HUA and/or gout. A total of 88 participants were enrolled, with the intervention group consuming probiotic yogurt daily for 12 weeks. The results showed a significant reduction in serum UA levels in the intervention group compared to the placebo group, with no serious adverse events observed (Yamanaka et al. 2019). In vitro studies have shown that *Ligilactobacillus salivarius* CECT 30632 can completely degrade inosine (100%) and guanosine (100%), and partially degrade uric acid (50%). A subsequent randomized, double‐blind, placebo‐controlled pilot clinical trial evaluated the effects of this probiotic strain in patients with HUA and gout. The results demonstrated that oral administration of the probiotic for 6 consecutive months significantly reduced serum UA levels and the frequency of gout attacks, with no serious adverse events reported (Rodriguez et al. 2023). These findings indicate good safety and tolerability, providing preliminary clinical evidence supporting the potential application of probiotics in HUA management.

Several strains such as *Lactobacillus* DM9218, *Lactobacillus Royce* TSR332, and *Lactobacillus fermentum* TSF331 have been shown to produce nucleoside hydrolases, which can degrade purine substances in food and reduce their intestinal absorption, thereby lowering serum UA levels (Wang et al. 2019). Li et al. (Vitetta and Gobe 2013) investigated the UA‐lowering effect of *Lactobacillus DM9218*, isolated from Chinese kimchi, and found that it was able to compete with rat intestinal epithelial cells for nucleosides in food. The cell‐free extract of this bacterium was able to degrade nucleosides, which was hypothesized to occur through purine nucleosidase activity, thus reducing UA levels. Wang et al. (2019) identified the inosine hydrolase gene in *Lactobacillus shortcombicus* DM9218 and expressed it in *Escherichia coli* . These results demonstrated that the modified *E. coli* had the ability to degrade inosine, further supporting the potential of probiotics in managing UA levels.

Therefore, probiotics can absorb nucleosides by competing with intestinal epithelial cells and degrade purine substances in the human intestinal tract by utilizing self‐produced purine nucleosidase. This process reduces the nucleoside content in the intestinal tract and inhibits the intestinal absorption of nucleosides, ultimately leading to a reduction in UA levels. Such competitive and enzymatic actions suggest a promising microbial mechanism for mitigating purine load and managing HUA.

### 7.3 Regulation of Gut Microbiome Homeostasis and Restoration of the Intestinal Barrier

The gut microbiota constitutes the largest and most complex micro‐ecological community within the human body, playing an indispensable role in preserving host health and physiological balance (Ma et al. 2019; Xiong et al. 2022). This intricate system comprises more than 1500 microbial species, including not only bacteria but also viruses and other microorganisms. The intestinal barrier, meanwhile, serves as a multifaceted defense mechanism, safeguarding the gastrointestinal tract from both endogenous and exogenous threats. It consists of physical, chemical, immune, and microbial elements, each performing distinct protective functions. Any disruption of the gut barrier has been associated with the onset of a range of diseases, such as irritable bowel syndrome, nonalcoholic fatty liver disease (NAFLD), type 2 diabetes, insulin resistance, and inflammatory bowel diseases (Chang et al. 2025; König et al. 2016; Turner 2009). The integrity and composition of gut microbiota are intimately linked to barrier function and the progression of these disorders. A balanced microbial community helps inhibit pathogenic invasion and translocation, enhances mucosal barrier strength, and limits toxin uptake, thereby contributing to intestinal health (Paolella et al. 2014). Furthermore, it supports immune function by stimulating the secretion of antimicrobial agents (Mills et al. 2019). Conversely, an imbalance in the gut microbiota, known as dysbiosis and 111, has been demonstrated to increase vulnerability to hypertension, gastrointestinal disorders, cardiovascular and metabolic diseases, as well as neuropsychiatric conditions such as anxiety, depression, and even cancer (Afzaal et al. 2022).

The functional balance of gut microbiome not only supports barrier integrity but also profoundly influences the host's metabolism of a wide range of endogenous compounds, including UA. One‐third of UA in the human body is excreted through the intestine and is influenced by the composition of the gut microbiota (Yin et al. 2022). Research has shown that maintaining or restoring a healthy microbiota can help modulate UA levels, partly through the action of specific enzymes such as urease and allantoinase produced by certain gut microbes. These enzymatic processes convert UA into metabolites that are more easily utilized or excreted by the host (Han et al. 2020; Méndez‐Salazar and Martínez‐Nava 2022; Ramazzina et al. 2010; Wu et al. 2021). Therefore, strategies that regulate the balance of the gut microbiota not only help restore the intestinal barrier but also play a critical role in the management of metabolic processes and in preventing an accumulation of metabolic waste products.

Transplantation of feces from HUA rats into normal rats resulted in a significant increase in serum UA levels in the normal rats, along with marked changes in the diversity and abundance of the gut microbiome in the HUA rats (Cheng et al. 2022). This suggests a potential link between gut microbiome and HUA. Microbiomic studies comparing the fecal flora characteristics of patients with gout and healthy individuals have shown that gout patients have lower flora abundance and diversity, with a significant increase in pathogenic bacteria such as *S. danielsii* and *Corynebacterium*. In a study of fecal transplants in HUA mice, it was found that fecal transplants restored the abundance of genera like those of thick‐walled bacteria, those of *Bacteroidetes*, and *Aspergillus*, demonstrating the modulatory effect of gut microbiome on HUA (Han et al. 2020). *L. paracasei* X11 increased the abundance of *Faecalibaculum* in the intestines of HUA mice, reduced the relative abundance of *Bacteroides* and *Aspergillus* species, and restored the normal *Firmicutes*‐to‐*Bacteroidetes* ratio to *Bacteroides* abundance. *L. fermentum* JL‐3 improved the structural and functional alterations of the gut microbiome in mice induced by UA, maintaining a stable state of the intestinal microbial community, which helped alleviate HUA (Wu et al. 2021). Additionally, a macrogenomic analysis of fecal samples from gout patients and healthy individuals showed that the abundance of SCFA‐producing strains was higher in healthy individuals than in patients with gout (Cheng et al. 2022; Yuan et al. 2022). The ethical feasibility of FMT for human HUA requires rigorous donor screening, long‐term risk monitoring, and informed consent, whereas clinical feasibility is constrained by the lack of standardized protocols, host microbiota heterogeneity, and competition with existing therapies. Although animal studies demonstrate its potential to modulate the microbiota, human application must address ethical concerns about the irreversibility of microecological intervention and validate therapeutic consistency and cost‐effectiveness in clinical trials.

HUA is also associated with intestinal permeability. Excessive intake of fructose has been shown to inhibit the expression of intestinal tight junction proteins, leading to dysregulation of the gut microbiome. This triggers inflammation and increases intestinal permeability, allowing inflammatory factors and toxic metabolites, such as LPS, to pass from the intestinal lumen into the portal vein. This can activate inflammatory factors and trigger related inflammation (Cho et al. 2021; Softic et al. 2018). Lv et al. (2020) found that HUA mice exhibited pro‐inflammatory effects because of dysregulated intestinal immunity and suggested that the combined disruption of intestinal immunity and intestinal ecology impaired the intestinal barrier, allowing microorganisms to enter from the circulatory system. Therefore, it is inferred from animal models that gut flora and gut barrier are closely related to HUA.

Treatment with *L. paracasei* MJM60396 significantly increased the expression of intestinal epithelial ZO‐1 and occludin, thereby protecting the intestinal barrier (Yamada et al. 2016). *Lactobacillus* species have been shown to promote tight junction protein expression and activate the Toll‐like receptor 2 signaling pathway (Karczewski et al. 2010). Furthermore, *L. rhamnosus* GG was found to produce P40 and P75 soluble proteins, repairing the damage to tight junction function and barrier integrity in Caco‐2 cells caused by hydrogen peroxide through the mitogen‐activated protein kinase pathway. This intervention reduced UA permeability and attenuated inflammatory responses (Seth et al. 2008). After intervention with *Akkermansia muciniphila* , the expression of intestinal tight junction proteins—claudin, occludin, and ZO‐1—was significantly increased by 70.35%, 179.16%, and 155.02%, respectively, compared to the model group mice. Additionally, after pasteurization of *A. muciniphila* , the expression of these proteins increased by 56.48%, 93.52%, and 66.09%, further reducing intestinal permeability and inhibiting the permeability of inflammatory factors (Zhang, Liu, et al. 2022).

These studies indicate that probiotics can regulate UA levels by modulating the diversity of the gut microbiome, adjusting the ratio of the gut microbiome, and repairing the intestinal barrier in HUA animals. Probiotics have the potential to serve as the basis for the early diagnosis of HUA, and a better understanding of the relationship between the gut microbiome and HUA can provide a theoretical foundation for the dietary treatment of HUA. However, further research is needed to investigate the underlying mechanisms by which probiotics regulate the gut microbiome and repair the intestinal barrier to reduce UA levels.

### 7.4 Impact on UA Excretion

Transporter proteins, such as OAT1, OAT3, multidrug resistance protein (MRP) 2, MRP4, nicotinate phosphoribosyl transferase 1, ABCG2, and others, have not yet been fully identified in relation to UA excretion (Table 3) (Lu et al. 2017). The effect of probiotics on UA excretion has not been fully explored. However, research suggests that probiotics may affect UA excretion by influencing the expression of urate transporters, promoting the expression of urate excretion transporter proteins, and inhibiting the expression of urate reabsorption proteins. Additionally, probiotics may directly degrade UA, converting it into more soluble substances such as allantoin, which facilitates increased UA excretion and reduced serum UA levels (Zhang et al. 2021).

**TABLE 3:** The role of transfer proteins.

Protein	Primary expression site	Role in uric acid handling	References
Organic Anion Transporter 1 (OAT1)	Kidney proximal tubule (basolateral membrane of tubular epithelial cells)	Uptakes urate from blood (interstitium) into renal tubular cells via urate–dicarboxylate exchange, facilitating urate secretion into the urine. Knockout of OAT1 in mice modestly reduces urate excretion, indicating its role in renal urate secretion	Sun et al. (2021)
Organic Anion Transporter 3 (OAT3)	Kidney proximal tubule (basolateral membrane); also in other tissues (e.g., choroid plexus) at lower levels	Functions similarly to OAT1, mediating uptake of urate (and other organic anions) from blood into renal proximal tubule cells in exchange for dicarboxylates. By importing urate into the cell, OAT3 enables subsequent apical efflux into urine. OAT3 knockout also slightly decreases urate excretion, confirming its role in net urate secretion	Sun et al. (2021)
Multidrug Resistance–Associated Protein 2 (MRP2)	Liver hepatocytes (canalicular membrane) and intestinal enterocytes (brush border); also in the kidney proximal tubule (apical brush‐border membrane)	ATP‐dependent efflux pump for organic anions. MRP2 can transport conjugated anions (e.g., bilirubin glucuronides, drug metabolites) into bile or urine. In vitro studies show MRP2 can transport urate, suggesting it may contribute to urate excretion into bile or urine. However, in vivo, its role in renal urate secretion appears limited or redundant (no clear decrease in urate excretion has been observed with MRP2 dysfunction)	Chai et al. (2015), Chung and Kim (2021)
Multidrug Resistance–Associated Protein 4 (MRP4)	Kidney proximal tubule (apical membrane); also in other tissues (e.g., apical membrane of colon, liver, and blood–brain barrier)	An ATP‐driven efflux transporter that exports urate and other organic anions across the apical membrane. MRP4 in the renal tubule cells pumps urate into the urine, acting as a unidirectional urate efflux pump for urinary excretion. It likewise may contribute to intestinal secretion of urate. In vitro, human MRP4 transports urate (and cyclic nucleotides, drug metabolites, etc.) efficiently. In MRP4‐deficient mice, urinary urate export is diminished (though compensatory mechanisms exist)	Benn et al. (2018), Tanner et al. (2017)
ATPBinding Cassette G2 (ABCG2)	Intestinal epithelium (apical membrane, especially colon) and kidney proximal tubule (apical lumenal membrane). Also expressed in other barrier tissues: e.g., liver canalicular membrane, placental syncytiotrophoblast, blood–brain barrier	High‐capacity urate exporter that actively secretes uric acid out of cells. In the gut, ABCG2 mediates direct intestinal excretion of urate into feces (accounting for a significant fraction of urate elimination). In the kidney, ABCG2 on proximal tubule cells transports urate into the urine (complementing renal secretion). ABCG2 thus provides an alternative route for uric acid disposal (“extrarenal” excretion via intestine) and works in tandem with renal transporters to prevent urate accumulation	Benn et al. (2018), Eckenstaler and Benndorf (2021)
Glucose Transporter 9 (GLUT9)	Primarily expressed in the liver and kidney, especially abundant in the epithelial cells of the renal proximal tubules	Functions as a basolateral uric acid efflux transporter in proximal tubular cells, transporting intracellular uric acid into the blood, thus mediating uric acid reabsorption. Kidney‐specific deletion of GLUT9 leads to increased uric acid excretion and elevated urine UA/creatinine ratio	Auberson et al. (2018), Chung and Kim (2021)
Urate Transporter 1 (URAT1)	Primarily expressed in the epithelial cells of the renal proximal tubules. It is localized on the apical brush border membrane of these cells and serves as one of the main transporters responsible for uric acid reabsorption in the renal tubules	URAT1 mediates the reabsorption of filtered uric acid from the tubular lumen into cells via an organic anion/uric acid exchange mechanism. It is the predominant transporter on the apical side of the proximal tubule. Loss of URAT1 impairs uric acid reabsorption, leading to increased urinary uric acid excretion. URAT1 and GLUT9 work synergistically—URAT1 is localized apically, and GLUT9 basolaterally—together facilitating the transport of uric acid from the urine back into the bloodstream	Chung and Kim (2021), Halperin Kuhns and Woodward (2021)

An increasing number of probiotic strains, which are able to regulate host metabolism and prevent chronic diseases without harmful side effects, have been characterized (Kuo et al. 2021; Li et al. 2022). For instance, the probiotic *Lactobacillus ingluviei* has been shown to influence UA excretion by affecting the expression of ABCG2, a UA transporter protein; *Lactobacillus ingluviei* is informative in its mechanistic studies (Angelakis et al. 2012). *L. rhamnosus* BFE5264 and *L. plantarum* NR74 upregulated the expression of ABCG1 and ATP‐binding cassette subfamily A member 1 at the cellular level (Yoon et al. 2013). Pasteurization of *A. muciniphila* resulted in a 63.84% and 72.81% reduction in the expression of URAT1 and GLUT9, respectively, and a 146.17% increase in the expression of ABCG2 (Zhang, Liu, et al. 2022). *L. paracasei* MJM60396 was also found to significantly increase the expression of urate excretion transporter proteins OAT1 mRNA and OAT3 mRNA while decreasing the expression of urate reabsorption proteins URAT1 mRNA and GLUT9 mRNA in the kidneys of mice, thereby enhancing UA excretion (Lee et al. 2022; Yamada et al. 2016). Similarly, *L. rhamnosus* Fmb14 inhibited the expression of URAT1 in the kidney and promoted the expression of ABCG2 in the colon (Zhao, Chen, et al. 2022).

Yasiri and Seubsasana (2020) found that *L. fermentum* SF121 directly degraded UA, facilitating its excretion and decreasing the activity of XOD, collectively lowering serum UA levels in mice. Additionally, *L. fermentum* JL‐3 was found to directly degrade UA, as its byproducts contained allantoin, suggesting that this bacterium may produce uricase. *L. plantarum* Dad‐13, *L. plantarum* Mut‐7, and *Lactobacillus casei* OL‐5 produce urease, which maintains activity under appropriate environmental conditions (Fadda et al. 2010; Handayani et al. 2018). However, it is important to note that humans naturally lack uricase, the enzyme responsible for converting uric acid to allantoin, which may pose challenges for the direct clinical translation of uricase‐producing probiotics. Although microbial degradation of UA into allantoin represents a promising strategy, its long‐term efficacy, safety, and metabolic consequences in humans require further investigation. Specifically, it remains to be clarified whether exogenous microbial uricase activity can function effectively in the human gut environment and whether the resulting allantoin can be safely metabolized or excreted without adverse effects. Therefore, although the ability of certain Lactobacillus strains to degrade UA is encouraging, future studies should focus on validating these findings in human subjects and exploring the regulatory pathways involved.

These findings indicate that some probiotics can directly break down UA, likely through the production of uricase, converting UA into soluble allantoin for excretion from the body. Furthermore, probiotics can reduce UA levels by inhibiting the expression of urate reabsorption proteins and promoting the expression of urate excretion transporter proteins. However, the specific mechanisms through which probiotics influence URAT expression remain unclear and warrant further investigation (Fujita et al. 2023; Vázquez‐Ávila et al. 2018). In this regard, hypothetical pathways could be proposed: probiotics may regulate URAT gene expression through metabolites such as SCFAs, or indirectly influence its activity by reshaping the gut microbiota structure. Additionally, ongoing research in this field is exploring interactions between specific strains (e.g., *Lactobacillus reuteri* , *Bifidobacterium*) and URAT subtypes (URAT1, GLUT9, etc.), which offer potential clues for mechanistic elucidation (Kuo et al. 2021).

## 8 Conclusions and Future Insights

The gut microbiome, which colonizes the human gut, plays a crucial role in the onset and progression of HUA. Changes in the composition and metabolism of the gut microbiota result in abnormalities of uric acid degradation, increasing uric acid generation. Therefore, exploring disease mechanisms and therapeutic targets through the lens of the gut microbiome has become a key focus of global research. Current research employs specific probiotic strains (e.g., *Lactobacillus plantarum* ) to promote SCFA production for UA synthesis inhibition and microbiota structure regulation, or utilizes high‐fiber/polyphenol diets to reshape gut microecology for UA metabolism improvement, whereas fecal microbiota transplantation explores restoring dysbiotic intestinal environments with healthy donor microbiota to intervene in HUA. These approaches focus on strategies like probiotic colonization, diet‐induced microbiota metabolic reprogramming, and whole‐microbiota transplantation to target the composition and function of gut microbiota, thereby influencing UA production, excretion, and systemic inflammatory status to intervene in HUA progression.

This review systematically analyzes the multifaceted roles of the gut microbiota in the pathogenesis of HUA, highlighting current research on its involvement in reducing UA production, promoting purine degradation, regulating amino acid transport, and enhancing UA excretion. Probiotic intervention has emerged as a promising adjunctive therapy because of its good safety profile and high patient acceptability. However, most existing studies are based on animal models, and clinical evidence remains limited. Moreover, the efficacy of probiotics may vary depending on specific strains and the host's baseline gut microbiota composition. Therefore, future research should prioritize large‐scale, long‐term clinical trials in HUA populations to evaluate the sustained effects of probiotics on serum UA levels and elucidate their underlying mechanisms. In parallel, studies utilizing humanized mouse models or non‐human primates are warranted to better mimic the human gut environment and enhance the translational relevance of findings. From a broader perspective, deepening our understanding of the regulatory role of the gut microbiome in HUA may not only facilitate the development of novel, safe, and targeted therapies, but also offer theoretical and practical insights for the prevention and management of related diseases such as gout and chronic kidney disease.

## Author Contributions

**Junyu Yang:** conceptualization (equal), visualization (equal), writing – original draft (equal), writing – review and editing (equal). **Jiali Chen:** visualization (equal), writing – original draft (equal), writing – review and editing (equal). **Zhenmin Liu:** supervision (equal), writing – review and editing (equal). **Yezhi Qu:** writing – review and editing (equal). **Xiqing Yue:** writing – original draft (equal), writing – review and editing (equal). **Bo Yuan:** conceptualization (equal), funding acquisition (equal), investigation (equal), project administration (equal), supervision (equal), writing – review and editing (equal). **Mohan Li:** supervision (equal), writing – original draft (equal), writing – review and editing (equal).

## Conflicts of Interest

The authors declare no conflicts of interest.

## Data Availability

No new data were generated or analyzed in this review. Data supporting the findings of this study are available in the referenced works.

## References

[fsn370982-bib-0001] Afzaal, M. , F. Saeed , Y. A. Shah , et al. 2022. “Human Gut Microbiota in Health and Disease: Unveiling the Relationship.” Frontiers in Microbiology 13: 999001. 10.3389/fmicb.2022.999001. 36225386 PMC9549250

[fsn370982-bib-0002] Agus, A. , K. Clément , and H. Sokol . 2021. “Gut Microbiota‐Derived Metabolites as Central Regulators in Metabolic Disorders.” Gut 70, no. 6: 1174–1182. 10.1136/gutjnl-2020-323071. 33272977 PMC8108286

[fsn370982-bib-0003] Ames, B. N. , R. Cathcart , E. Schwiers , and P. Hochstein . 1981. “Uric Acid Provides an Antioxidant Defense in Humans Against Oxidant‐ and Radical‐Caused Aging and Cancer: A Hypothesis.” Proceedings of the National Academy of Sciences of the United States of America 78, no. 11: 6858–6862. 10.1073/pnas.78.11.6858. 6947260 PMC349151

[fsn370982-bib-0004] Angelakis, E. , D. Bastelica , A. Ben Amara , et al. 2012. “An Evaluation of the Effects of *Lactobacillus ingluviei* on Body Weight, the Intestinal Microbiome and Metabolism in Mice.” Microbial Pathogenesis 52, no. 1: 61–68. 10.1016/j.micpath.2011.10.004. 22020311

[fsn370982-bib-0005] Angelucci, F. , K. Cechova , J. Amlerova , and J. Hort . 2019. “Antibiotics, Gut Microbiota, and Alzheimer's Disease.” Journal of Neuroinflammation 16: 108. 10.1186/s12974-019-1494-4. 31118068 PMC6530014

[fsn370982-bib-0006] Arakawa, H. , N. Amezawa , Y. Kawakatsu , and I. Tamai . 2020. “Renal Reabsorptive Transport of Uric Acid Precursor Xanthine by URAT1 and GLUT9.” Biological & Pharmaceutical Bulletin 43, no. 11: 1792–1798. 10.1248/bpb.b20-00597. 33132325

[fsn370982-bib-0007] Armour, C. R. , S. Nayfach , K. S. Pollard , and T. J. Sharpton . 2019. “A Metagenomic Meta‐Analysis Reveals Functional Signatures of Health and Disease in the Human Gut Microbiome.” MSystems 4, no. 4: e00332‐18. 10.1128/mSystems.00332-18. 31098399 PMC6517693

[fsn370982-bib-0008] Auberson, M. , S. Stadelmann , C. Stoudmann , et al. 2018. “SLC2A9 (GLUT9) Mediates Urate Reabsorption in the Mouse Kidney.” Pflügers Archiv 470, no. 12: 1739–1751. 10.1007/s00424-018-2190-4. 30105595 PMC6224025

[fsn370982-bib-0009] Bai, M. , H. Liu , Y. Yan , et al. 2024. “Hydrolyzed Protein Formula Improves the Nutritional Tolerance by Increasing Intestinal Development and Altering Cecal Microbiota in Low‐Birth‐Weight Piglets.” Frontiers in Nutrition 11: 1439110. 10.3389/fnut.2024.1439110. 39555191 PMC11565607

[fsn370982-bib-0010] Bardin, T. , and P. Richette . 2017. “Impact of Comorbidities on Gout and Hyperuricaemia: An Update on Prevalence and Treatment Options.” BMC Medicine 15: 123. 10.1186/s12916-017-0890-9. 28669352 PMC5494879

[fsn370982-bib-0011] Benn, C. L. , P. Dua , R. Gurrell , et al. 2018. “Physiology of Hyperuricemia and Urate‐Lowering Treatments.” Frontiers in Medicine 5: 160. 10.3389/fmed.2018.00160. 29904633 PMC5990632

[fsn370982-bib-0012] Bian, M. , J. Wang , Y. Wang , et al. 2020. “Chicory Ameliorates Hyperuricemia via Modulating Gut Microbiota and Alleviating LPS/TLR4 Axis in Quail.” Biomedicine & Pharmacotherapy 131: 110719. 10.1016/j.biopha.2020.110719. 33152909

[fsn370982-bib-0013] Buzard, J. , C. Bishop , and J. H. Talbott . 1952. “Recovery in Humans of Intravenously Injected Isotopic Uric Acid.” Journal of Biological Chemistry 196, no. 1: 179–184. 12980954

[fsn370982-bib-0014] Cao, J. , T. Wang , Y. Liu , et al. 2023. “ *Lactobacillus fermentum* F40‐4 Ameliorates Hyperuricemia by Modulating the Gut Microbiota and Alleviating Inflammation in Mice.” Food & Function 14, no. 7: 3259–3268. 10.1039/d2fo03701g. 36928268

[fsn370982-bib-0015] Cao, J. Y. , Y. S. Bu , H. N. Hao , et al. 2022. “Effect and Potential Mechanism of *Lactobacillus plantarum* Q7 on Hyperuricemia *In Vitro* and *In Vivo* .” Frontiers in Nutrition 9: 954545. 10.3389/fnut.2022.954545. 35873427 PMC9298507

[fsn370982-bib-0016] Cao, J. Y. , Q. Q. Liu , H. N. Hao , et al. 2022. “ *Lactobacillus paracasei* X11 Ameliorates Hyperuricemia and Modulates Gut Microbiota in Mice.” Frontiers in Immunology 13: 940228. 10.3389/fimmu.2022.940228. 35874662 PMC9296831

[fsn370982-bib-0017] Cao, T. , X. Y. Li , T. Mao , et al. 2017. “Probiotic Therapy Alleviates Hyperuricemia in C57BL/6 Mouse Model.” Biomedical Research‐India 28, no. 5: 2244–2249.

[fsn370982-bib-0018] Chai, J. , S. Y. Cai , X. Liu , et al. 2015. “Canalicular Membrane MRP2/ABCC2 Internalization Is Determined by Ezrin Thr567 Phosphorylation in Human Obstructive Cholestasis.” Journal of Hepatology 63, no. 6: 1440–1448. 10.1016/j.jhep.2015.07.016. 26212029 PMC4686151

[fsn370982-bib-0019] Chang, G. , S. Tian , X. Luo , et al. 2025. “Hypoglycemic Effects and Mechanisms of Polyphenols From *Myrica rubra* Pomace in Type 2 Diabetes (Db/Db) Mice.” Molecular Nutrition & Food Research 69, no. 10: e202400523. 10.1002/mnfr.202400523. 40171790

[fsn370982-bib-0020] Chen, F. , Y. Wang , K. Wang , et al. 2023. “Effects of *Litsea cubeba* Essential Oil on Growth Performance, Blood Antioxidation, Immune Function, Apparent Digestibility of Nutrients, and Fecal Microflora of Pigs.” Frontiers in Pharmacology 14: 1166022. 10.3389/fphar.2023.1166022. 37465523 PMC10350539

[fsn370982-bib-0021] Chen, Y. , L. Y. Luo , S. S. Hu , R. Y. Gan , and L. Zeng . 2023. “The Chemistry, Processing, and Preclinical Anti‐Hyperuricemia Potential of Tea: A Comprehensive Review.” Critical Reviews in Food Science and Nutrition 63, no. 24: 7065–7090. 10.1080/10408398.2022.2040417. 35236179

[fsn370982-bib-0022] Cheng, Y. W. , J. M. Liu , and Z. X. Ling . 2022. “Short‐Chain Fatty Acids‐Producing Probiotics: A Novel Source of Psychobiotics.” Critical Reviews in Food Science and Nutrition 62, no. 28: 7929–7959. 10.1080/10408398.2021.1920884. 33955288

[fsn370982-bib-0023] Cho, Y. E. , D. K. Kim , W. Seo , B. Gao , S. H. Yoo , and B. J. Song . 2021. “Fructose Promotes Leaky Gut, Endotoxemia, and Liver Fibrosis Through Ethanol‐Inducible Cytochrome P450‐2E1‐Mediated Oxidative and Nitrative Stress.” Hepatology 73, no. 6: 2180–2195. 10.1002/hep.30652. 30959577 PMC6783321

[fsn370982-bib-0024] Choi, Y. J. , Y. Yoon , K. Y. Lee , et al. 2014. “Uric Acid Induces Endothelial Dysfunction by Vascular Insulin Resistance Associated With the Impairment of Nitric Oxide Synthesis.” FASEB Journal 28, no. 7: 3197–3204. 10.1096/fj.13-247148. 24652948

[fsn370982-bib-0025] Chu, Y. L. , S. L. Sun , Y. F. Huang , et al. 2021. “Metagenomic Analysis Revealed the Potential Role of Gut Microbiome in Gout.” npj Biofilms and Microbiomes 7, no. 1: 66. 10.1038/s41522-021-00235-2. 34373464 PMC8352958

[fsn370982-bib-0026] Chung, S. , and G. H. Kim . 2021. “Urate Transporters in the Kidney: What Clinicians Need to Know.” Electrolyte Blood Pressure 19, no. 1: 1–9. 10.5049/EBP.2021.19.1.1. 34290818 PMC8267069

[fsn370982-bib-0027] Crawley, W. T. , C. G. Jungels , K. R. Stenmark , and M. A. Fini . 2022. “U‐Shaped Association of Uric Acid to Overall‐Cause Mortality and Its Impact on Clinical Management of Hyperuricemia.” Redox Biology 51: 102271. 10.1016/j.redox.2022.102271. 35228125 PMC8889273

[fsn370982-bib-0028] Dalbeth, N. , H. K. Choi , L. A. B. Joosten , et al. 2019. “Gout.” Nature Reviews Disease Primers 5, no. 1: 69. 10.1038/s41572-019-0115-y. 31558729

[fsn370982-bib-0029] Ding, R. X. , M. H. Li , Y. T. Zou , et al. 2022. “Effect of Normal and Strict Anaerobic Fermentation on Physicochemical Quality and Metabolomics of Yogurt.” Food Bioscience 46: 101368. 10.1016/j.fbio.2021.101368.

[fsn370982-bib-0030] Do, M. H. , E. Lee , M. J. Oh , Y. Kim , and H. Y. Park . 2018. “High‐Glucose or ‐Fructose Diet Cause Changes of the Gut Microbiota and Metabolic Disorders in Mice Without Body Weight Change.” Nutrients 10, no. 6: 761. 10.3390/nu10060761. 29899272 PMC6024874

[fsn370982-bib-0031] Dong, L. , F. Dong , P. Guo , et al. 2025. “Gut Microbiota as a New Target for Hyperuricemia: A Perspective From Natural Plant Products.” Phytomedicine 138: 156402. 10.1016/j.phymed.2025.156402. 39874797

[fsn370982-bib-0032] Du, J. N. , N. Wang , D. H. Yu , et al. 2024. “Data Mining‐Guided Alleviation of Hyperuricemia by *Paeonia veitchii* Lynch Through Inhibition of Xanthine Oxidase and Regulation of Renal Urate Transporters.” Phytomedicine 124: 155305. 10.1016/j.phymed.2023.155305. 38176275

[fsn370982-bib-0033] Du, L. , Y. Zong , H. Li , et al. 2024. “Hyperuricemia and Its Related Diseases: Mechanisms and Advances in Therapy.” Signal Transduction and Targeted Therapy 9, no. 1: 212. 10.1038/s41392-024-01916-y. 39191722 PMC11350024

[fsn370982-bib-0034] Ebrahimpour‐Koujan, S. , P. Saneei , B. Larijani , and A. Esmaillzadeh . 2020. “Consumption of Sugar Sweetened Beverages and Dietary Fructose in Relation to Risk of Gout and Hyperuricemia: A Systematic Review and Meta‐Analysis.” Critical Reviews in Food Science and Nutrition 60, no. 1: 1–10. 10.1080/10408398.2018.1503155. 30277800

[fsn370982-bib-0035] Eckenstaler, R. , and R. A. Benndorf . 2021. “The Role of ABCG2 in the Pathogenesis of Primary Hyperuricemia and Gout‐An Update.” International Journal of Molecular Sciences 22, no. 13: 6678. 10.3390/ijms22136678. 34206432 PMC8268734

[fsn370982-bib-0036] Esche, J. , D. Krupp , G. B. M. Mensink , and T. Remer . 2020. “Estimates of Renal Net Acid Excretion and Their Relationships With Serum Uric Acid and Hyperuricemia in a Representative German Population Sample.” European Journal of Clinical Nutrition 74: 63–68. 10.1038/s41430-020-0688-2. 32873959

[fsn370982-bib-0037] Fadda, S. , P. Anglade , F. Baraige , et al. 2010. “Adaptive Response of *Lactobacillus sakei* 23K During Growth in the Presence of Meat Extracts: A Proteomic Approach.” International Journal of Food Microbiology 142, no. 1–2: 36–43. 10.1016/j.ijfoodmicro.2010.05.014. 20580114

[fsn370982-bib-0038] Frei, B. , R. Stocker , and B. N. Ames . 1989. “Antioxidant Defenses and Lipid Peroxidation in Human Blood Plasma.” Proceedings of the National Academy of Sciences of the United States of America 85, no. 24: 9748–9752. 10.1073/pnas.85.24.9748 PMC282858 3200852

[fsn370982-bib-0039] Fujita, K. , Q. N. Zhu , H. Arakawa , Y. Shirasaka , and I. Tamai . 2023. “Potentiation of the Uricosuric Effect of Dotinurad by Trans‐Inhibition of the Uric Acid Reabsorptive Transporter 1.” Drug Metabolism and Disposition 51, no. 11: 1527–1535. 10.1124/dmd.123.001412. 37643882

[fsn370982-bib-0040] Galozzi, P. , S. Bindoli , A. Doria , F. Oliviero , and P. Sfriso . 2021. “Autoinflammatory Features in Gouty Arthritis.” Journal of Clinical Medicine 10, no. 9: 1880. 10.3390/jcm10091880. 33926105 PMC8123608

[fsn370982-bib-0041] Giordano, C. , O. Karasik , K. King‐Morris , and A. Asmar . 2015. “Uric Acid as a Marker of Kidney Disease: Review of the Current Literature.” Disease Markers 2015: 382918. 10.1155/2015/382918. 26106252 PMC4461768

[fsn370982-bib-0042] Guo, Z. , J. C. Zhang , Z. L. Wang , et al. 2016. “Intestinal Microbiota Distinguish Gout Patients From Healthy Humans.” Scientific Reports 6: 20602. 10.1038/srep20602. 26852926 PMC4757479

[fsn370982-bib-0043] Halperin Kuhns, V. L. , and O. M. Woodward . 2021. “Urate Transport in Health and Disease.” Best Practice & Research. Clinical Rheumatology 35, no. 4: 101717. 10.1016/j.berh.2021.101717. 34690083 PMC8678298

[fsn370982-bib-0044] Hamada, T. , K. Ichida , M. Hosoyamada , et al. 2008. “Uricosuric Action of Losartan via the Inhibition of Urate Transporter 1 (URAT 1) in Hypertensive Patients.” American Journal of Hypertension 21, no. 10: 1157–1162. 10.1038/ajh.2008.245. 18670416

[fsn370982-bib-0045] Han, J. J. , X. F. Wang , S. S. Tang , et al. 2020. “Protective Effects of Tuna Meat Oligopeptides (TMOP) Supplementation on Hyperuricemia and Associated Renal Inflammation Mediated by Gut Microbiota.” FASEB Journal 34, no. 4: 5061–5076. 10.1096/fj.201902597RR. 32043638

[fsn370982-bib-0046] Han, T. S. , X. Meng , R. Q. Shan , et al. 2018. “Temporal Relationship Between Hyperuricemia and Obesity, and Its Association With Future Risk of Type 2 Diabetes.” International Journal of Obesity 42, no. 7: 1336–1344. 10.1038/s41366-018-0074-5. 29717279

[fsn370982-bib-0047] Handayani, I. , T. Utami , C. Hidayat , and E. S. Rahayu . 2018. “Screening of Lactic Acid Bacteria Producing Uricase and Stability Assessment in Simulated Gastrointestinal Conditions.” International Food Research Journal 25, no. 4: 1661–1667.

[fsn370982-bib-0048] Hisatome, I. , A. Ohtahara , T. Hamada , and K. Ogino . 2018. “Development of Guideline for the Management of Hyperuricemia and Gout in Japan 3rd Edition.” Gout and Nucleic Acid Metabolism 42, no. 2: 147–156.

[fsn370982-bib-0049] Hsu, C.‐L. , Y.‐H. Wang , C.‐S. Lin , et al. 2019. “Antiobesity and Uric Acid‐Lowering Effect of *Lactobacillus plantarum* GKM3 in High‐Fat‐Diet‐Induced Obese Rats.” Journal of the American College of Nutrition 38, no. 7: 623–632. 30794474 10.1080/07315724.2019.1571454

[fsn370982-bib-0050] Huang, Q. Q. , C. M. Harvey , A. R. Paterson , C. E. Cass , and J. D. Young . 1993. “Functional Expression of Na(+)‐Dependent Nucleoside Transport Systems of Rat Intestine in Isolated Oocytes of *Xenopus laevis* . Demonstration That Rat Jejunum Expresses the Purine‐Selective System N1 (Cif) and a Second, Novel System N3 Having Broad Specificity for Purine and Pyrimidine Nucleosides.” Journal of Biological Chemistry 268, no. 27: 20613–20619. 7690759

[fsn370982-bib-0051] Ichida, K. , H. Matsuo , T. Takada , et al. 2012. “Decreased Extra‐Renal Urate Excretion Is a Common Cause of Hyperuricemia.” Nature Communications 3: 764. 10.1038/ncomms1756. PMC3337984 22473008

[fsn370982-bib-0052] Ishii, Y. , S. Sugimoto , N. Izawa , T. Sone , K. Chiba , and K. Miyazaki . 2014. “Oral Administration of *Bifidobacterium breve* Attenuates UV‐Induced Barrier Perturbation and Oxidative Stress in Hairless Mice Skin.” Archives of Dermatological Research 306, no. 5: 467–473. 10.1007/s00403-014-1441-2. 24414333

[fsn370982-bib-0053] Jalal, D. I. , M. Chonchol , W. Chen , and G. Targher . 2013. “Uric Acid as a Target of Therapy in CKD.” American Journal of Kidney Diseases 61, no. 1: 134–146. 10.1053/j.ajkd.2012.07.021. 23058478 PMC3525781

[fsn370982-bib-0054] Jin, M. , F. Yang , I. Yang , et al. 2012. “Uric Acid, Hyperuricemia and Vascular Diseases.” Frontiers in Bioscience 17: 656–669. 10.2741/3950 PMC3247913 22201767

[fsn370982-bib-0055] Jung, S. W. , S. M. Kim , Y. G. Kim , S. H. Lee , and J. Y. Moon . 2020. “Uric Acid and Inflammation in Kidney Disease.” American Journal of Physiology. Renal Physiology 318, no. 6: F1327–F1340. 10.1152/ajprenal.00272.2019. 32223310

[fsn370982-bib-0056] Kang, E. H. , E. H. Park , A. Shin , J. S. Song , and S. C. Kim . 2021. “Cardiovascular Risk Associated With Allopurinol vs. Benzbromarone in Patients With Gout.” European Heart Journal 42, no. 44: 4578–4588. 10.1093/eurheartj/ehab619. 34508567 PMC8633759

[fsn370982-bib-0057] Karczewski, J. , F. J. Troost , I. Konings , et al. 2010. “Regulation of Human Epithelial Tight Junction Proteins by *Lactobacillus plantarum* In Vivo and Protective Effects on the Epithelial Barrier.” American Journal of Physiology. Gastrointestinal and Liver Physiology 298, no. 6: G851–G859. 10.1152/ajpgi.00327.2009. 20224007

[fsn370982-bib-0058] Kasahara, K. , R. L. Kerby , Q. Zhang , et al. 2023. “Gut Bacterial Metabolism Contributes to Host Global Purine Homeostasis.” Cell Host & Microbe 31, no. 6: 1038–1053.e1010. 10.1016/j.chom.2023.05.011. 37279756 PMC10311284

[fsn370982-bib-0059] Kilstrup, M. , K. Hammer , P. Ruhdal Jensen , and J. Martinussen . 2005. “Nucleotide Metabolism and Its Control in Lactic Acid Bacteria.” FEMS Microbiology Reviews 29, no. 3: 555–590. 10.1016/j.femsre.2005.04.006. 15935511

[fsn370982-bib-0060] Kolz, M. , T. Johnson , S. Sanna , et al. 2009. “Meta‐Analysis of 28,141 Individuals Identifies Common Variants Within Five New Loci That Influence Uric Acid Concentrations.” PLoS Genetics 5, no. 6: e1000504. 10.1371/journal.pgen.1000504. 19503597 PMC2683940

[fsn370982-bib-0061] König, J. , J. Wells , P. D. Cani , et al. 2016. “Human Intestinal Barrier Function in Health and Disease.” Clinical and Translational Gastroenterology 7, no. 10: e196. 10.1038/ctg.2016.54. 27763627 PMC5288588

[fsn370982-bib-0062] Köttgen, A. , E. Albrecht , A. Teumer , et al. 2013. “Genome‐Wide Association Analyses Identify 18 New Loci Associated With Serum Urate Concentrations.” Nature Genetics 45, no. 2: 145–154. 10.1038/ng.2500. 23263486 PMC3663712

[fsn370982-bib-0063] Kuo, Y. W. , S. H. Hsieh , J. F. Chen , et al. 2021. “ *Lactobacillus reuteri* TSR332 and *Lactobacillus fermentum* TSF331 Stabilize Serum Uric Acid Levels and Prevent Hyperuricemia in Rats.” PeerJ 9: e11209. 10.7717/peerj.11209. 33986988 PMC8101448

[fsn370982-bib-0064] Lee, Y. , P. Werlinger , J. W. Suh , and J. H. Cheng . 2022. “Potential Probiotic *Lacticaseibacillus paracasei* MJM60396 Prevents Hyperuricemia in a Multiple Way by Absorbing Purine, Suppressing Xanthine Oxidase and Regulating Urate Excretion in Mice.” Microorganisms 10, no. 5: 851. 10.3390/microorganisms10050851. 35630296 PMC9146106

[fsn370982-bib-0065] Li, D. , S. Yuan , Y. Deng , et al. 2023. “The Dysregulation of Immune Cells Induced by Uric Acid: Mechanisms of Inflammation Associated With Hyperuricemia and Its Complications.” Frontiers in Immunology 14: 1282890. 10.3389/fimmu.2023.1282890. 38053999 PMC10694226

[fsn370982-bib-0066] Li, H. , Y. Zhou , L. Liao , et al. 2022. “Pharmacokinetics Effects of Chuanxiong Rhizoma on Warfarin in Pseudo Germ‐Free Rats.” Frontiers in Pharmacology 13: 1022567. 10.3389/fphar.2022.1022567. 36686675 PMC9849362

[fsn370982-bib-0067] Li, H. Y. , D. D. Zhou , R. Y. Gan , et al. 2021. “Effects and Mechanisms of Probiotics, Prebiotics, Synbiotics, and Postbiotics on Metabolic Diseases Targeting Gut Microbiota: A Narrative Review.” Nutrients 13, no. 9: 3211. 10.3390/nu13093211. 34579087 PMC8470858

[fsn370982-bib-0068] Li, L. , T. Li , X. Liang , et al. 2025. “A Decrease in *Flavonifractor plautii* and Its Product, Phytosphingosine, Predisposes Individuals With Phlegm‐Dampness Constitution to Metabolic Disorders.” Cell Discovery 11, no. 1: 25. 10.1038/s41421-025-00789-x. 40097405 PMC11914097

[fsn370982-bib-0069] Li, L. J. , Y. P. Zhang , and C. C. Zeng . 2020. “Update on the Epidemiology, Genetics, and Therapeutic Options of Hyperuricemia.” American Journal of Translational Research 12, no. 7: 3167–3181. 32774692 PMC7407685

[fsn370982-bib-0070] Li, M. , Q. L. Li , R. Abdlla , J. L. Chen , X. Q. Yue , and S. Y. Quek . 2023. “Donkey Whey Proteins Ameliorate Dextran Sulfate Sodium‐Induced Ulcerative Colitis in Mice by Downregulating the S100A8‐TRAF6‐NF‐? B Axis‐Mediated Inflammatory Response.” Food Science and Human Wellness 12, no. 5: 1809–1819. 10.1016/j.fshw.2023.02.045.

[fsn370982-bib-0071] Li, M. , D. B. Yang , L. Mei , L. Yuan , A. Xie , and J. L. Yuan . 2014. “Screening and Characterization of Purine Nucleoside Degrading Lactic Acid Bacteria Isolated From Chinese Sauerkraut and Evaluation of the Serum Uric Acid Lowering Effect in Hyperuricemic Rats.” PLoS One 9, no. 9: e105577. 10.1371/journal.pone.0105577. 25184445 PMC4153548

[fsn370982-bib-0072] Li, S. , S. Sanna , A. Maschio , et al. 2007. “The GLUT9 Gene Is Associated With Serum Uric Acid Levels in Sardinia and Chianti Cohorts.” PLoS Genetics 3, no. 11: e194. 10.1371/journal.pgen.0030194. 17997608 PMC2065883

[fsn370982-bib-0073] Liang, M. T. , J. K. Liu , W. J. Chen , et al. 2022. “Diagnostic Model for Predicting Hyperuricemia Based on Alterations of the Gut Microbiome in Individuals With Different Serum Uric Acid Levels.” Frontiers in Endocrinology 13: 925119. 10.3389/fendo.2022.925119. 36237183 PMC9553226

[fsn370982-bib-0074] Lima, W. G. , M. E. S. Martins‐Santos , and V. E. Chaves . 2015. “Uric Acid as a Modulator of Glucose and Lipid Metabolism.” Biochimie 116: 17–23. 10.1016/j.biochi.2015.06.025. 26133655

[fsn370982-bib-0075] Liu, M. Y. , X. N. Wu , H. L. Chen , et al. 2024. “Exploring the Mechanism and Inhibitory Effect of Robinin on Xanthine Oxidase Using Multi‐Spectroscopy and Molecular Dynamics Simulation Methods.” Journal of Molecular Liquids 408: 125373. 10.1016/j.molliq.2024.125373.

[fsn370982-bib-0076] Liu, X. , Q. Lv , H. Ren , et al. 2020. “The Altered Gut Microbiota of High‐Purine‐Induced Hyperuricemia Rats and Its Correlation With Hyperuricemia.” PeerJ 8: e8664. 10.7717/peerj.8664. 32185104 PMC7061907

[fsn370982-bib-0077] Lu, H. , Z. Q. Lu , X. Li , et al. 2017. “Interactions of 172 Plant Extracts With Human Organic Anion Transporter 1 (SLC22A6) and 3 (SLC22A8): A Study on Herb‐Drug Interactions.” PeerJ 5: e3333. 10.7717/peerj.3333. 28560096 PMC5446775

[fsn370982-bib-0078] Lu, J. , N. Dalbeth , H. Y. Yin , C. G. Li , T. R. Merriman , and W. H. Wei . 2019. “Mouse Models for Human Hyperuricaemia: A Critical Review.” Nature Reviews Rheumatology 15, no. 7: 413–426. 10.1038/s41584-019-0222-x. 31118497

[fsn370982-bib-0079] Luyun, C. , L. Liping , C. Ailing , X. Jing , L. I. Jianrong , and S. Lin . 2018. Research Progress of Purine Content Distribution in Food Technology.

[fsn370982-bib-0080] Lv, Q. L. , D. X. Xu , X. Z. Zhang , et al. 2020. “Association of Hyperuricemia With Immune Disorders and Intestinal Barrier Dysfunction.” Frontiers in Physiology 11: 524236. 10.3389/fphys.2020.524236. 33329010 PMC7729003

[fsn370982-bib-0081] Ma, C. J. , G. J. Cheng , Z. M. Liu , G. Y. Gong , and Z. J. Chen . 2016. “Determination of the Essential Nutrients Required for Milk Fermentation by *Lactobacillus plantarum* .” LWT – Food Science and Technology 65: 884–889. 10.1016/j.lwt.2015.09.003.

[fsn370982-bib-0082] Ma, Q. , Y. Li , P. Li , et al. 2019. “Research Progress in the Relationship Between Type 2 Diabetes Mellitus and Intestinal Flora.” Biomedicine & Pharmacotherapy 117: 109138. 10.1016/j.biopha.2019.109138. 31247468

[fsn370982-bib-0083] Maiuolo, J. , F. Oppedisano , S. Gratteri , C. Muscoli , and V. Mollace . 2023. “Regulation of Uric Acid Metabolism and Excretion.” International Journal of Cardiology 213: 8–14. 10.1016/j.ijcard.2023.131126. 26316329

[fsn370982-bib-0084] Major, T. J. , R. K. Topless , N. Dalbeth , and T. R. Merriman . 2018. “Evaluation of the Diet Wide Contribution to Serum Urate Levels: Meta‐Analysis of Population Based Cohorts.” BMJ (Clinical Research Ed.) 363: k3951. 10.1136/bmj.k3951. PMC6174725 30305269

[fsn370982-bib-0085] Mancikova, A. , V. Krylov , O. Hurba , et al. 2016. “Functional Analysis of Novel Allelic Variants in URAT1 and GLUT9 Causing Renal Hypouricemia Type 1 and 2.” Clinical and Experimental Nephrology 20, no. 4: 578–584. 10.1007/s10157-015-1186-z. 26500098

[fsn370982-bib-0086] Martens, K. L. , P. R. Khalighi , S. Li , et al. 2020. “Comparative Effectiveness of Rasburicase Versus Allopurinol for Cancer Patients With Renal Dysfunction and Hyperuricemia.” Leukemia Research 89: 106298. 10.1016/j.leukres.2020.106298. 31945598

[fsn370982-bib-0087] Martinez‐Quintana, E. , A. Tugores , and F. Rodriguez‐Gonzalez . 2016. “Serum Uric Acid Levels and Cardiovascular Disease: The Gordian Knot.” Journal of Thoracic Disease 8, no. 11: E1462–E1466. 10.21037/jtd.2016.11.39. 28066631 PMC5179380

[fsn370982-bib-0088] Massy, Z. A. , and T. B. Drueke . 2021. “Diet‐Microbiota Interaction and Kidney Disease Progression.” Kidney International 99, no. 4: 797–800. 10.1016/j.kint.2020.11.006. 33245991

[fsn370982-bib-0089] Matsuo, H. , E. Ishikawa , H. Machida , et al. 2020. “Efficacy of Xanthine Oxidase Inhibitor for Chronic Kidney Disease Patients With Hyperuricemia.” Clinical and Experimental Nephrology 24, no. 4: 307–313. 10.1007/s10157-019-01829-z. 31845065

[fsn370982-bib-0090] Méndez‐Salazar, E. O. , and G. A. Martínez‐Nava . 2022. “Uric Acid Extrarenal Excretion: The Gut Microbiome as an Evident Yet Understated Factor in Gout Development.” Rheumatology International 42, no. 3: 403–412. 10.1007/s00296-021-05007-x. 34586473

[fsn370982-bib-0091] Meng, Y. , Y. Hu , M. Wei , et al. 2023. “Amelioration of Hyperuricemia by *Lactobacillus acidophilus* F02 With Uric Acid‐Lowering Ability via Modulation of NLRP3 Inflammasome and Gut Microbiota Homeostasis.” Journal of Functional Foods 111: 105903. 10.1016/j.jff.2023.105903.

[fsn370982-bib-0092] Merriman, T. R. , and N. J. J. b. s. Dalbeth . 2011. “The Genetic Basis of Hyperuricaemia and Gout.” Joint, Bone, Spine 78, no. 1: 35–40. 20472486 10.1016/j.jbspin.2010.02.027

[fsn370982-bib-0093] Mills, S. , C. Stanton , J. A. Lane , G. J. Smith , and R. P. Ross . 2019. “Precision Nutrition and the Microbiome, Part I: Current State of the Science.” Nutrients 11, no. 4: 923. 10.3390/nu11040923. 31022973 PMC6520976

[fsn370982-bib-0094] Nakagawa, T. , M. A. Lanaspa , and R. J. Johnson . 2019. “The Effects of Fruit Consumption in Patients With Hyperuricaemia or Gout.” Rheumatology 58, no. 7: 1133–1141. 10.1093/rheumatology/kez128. 31004140

[fsn370982-bib-0095] Nakai, K. , M. B. Kadiiska , J.‐J. Jiang , K. Stadler , and R. P. Mason . 2006. “Free Radical Production Requires Both Inducible Nitric Oxide Synthase and Xanthine Oxidase in LPS‐Treated Skin.” Proceedings of the National Academy of Sciences of the United States of America 103, no. 12: 4616–4621. 10.1073/pnas.0510352103. 16537416 PMC1450220

[fsn370982-bib-0096] Nielsen, S. M. , K. Zobbe , L. E. Kristensen , and R. Christensen . 2018. “Nutritional Recommendations for Gout: An Update From Clinical Epidemiology.” Autoimmunity Reviews 17, no. 11: 1090–1096. 10.1016/j.autrev.2018.05.008. 30213692

[fsn370982-bib-0097] Nomura, J. , N. Busso , A. Ives , et al. 2013. “Febuxostat, an Inhibitor of Xanthine Oxidase, Suppresses Lipopolysaccharide‐Induced MCP‐1 Production via MAPK Phosphatase‐1‐Mediated Inactivation of JNK.” PLoS One 8, no. 9: e75527. 10.1371/journal.pone.0075527. 24086554 PMC3783396

[fsn370982-bib-0098] O'Dell, J. R. , M. T. Brophy , M. H. Pillinger , et al. 2022. “Comparative Effectiveness of Allopurinol and Febuxostat in Gout Management.” NEJM Evidence 1, no. 3 : evidoa2100028. 10.1056/evidoa2100028. PMC9012032 35434725

[fsn370982-bib-0099] Pan, L. B. , P. Han , S. R. Ma , et al. 2020. “Abnormal Metabolism of Gut Microbiota Reveals the Possible Molecular Mechanism of Nephropathy Induced by Hyperuricemia.” Acta Pharmaceutica Sinica B 10, no. 2: 249–261. 10.1016/j.apsb.2019.10.007. 32082971 PMC7016297

[fsn370982-bib-0100] Paolella, G. , C. Mandato , L. Pierri , M. Poeta , M. Di Stasi , and P. Vajro . 2014. “Gut‐Liver Axis and Probiotics: Their Role in Non‐Alcoholic Fatty Liver Disease.” World Journal of Gastroenterology 20, no. 42: 15518–15531. 10.3748/wjg.v20.i42.15518. 25400436 PMC4229517

[fsn370982-bib-0101] Park, B. , H. A. Lee , S. H. Lee , et al. 2017. “Association Between Serum Levels of Uric Acid and Blood Pressure Tracking in Childhood.” American Journal of Hypertension 30, no. 7: 713–718. 10.1093/ajh/hpx037. 28338917

[fsn370982-bib-0102] Ramazzina, I. , R. Costa , L. Cendron , et al. 2010. “An Aminotransferase Branch Point Connects Purine Catabolism to Amino Acid Recycling.” Nature Chemical Biology 6, no. 11: 801–806. 10.1038/nchembio.445. 20852637

[fsn370982-bib-0103] Rao, L. , B. Dong , Y. Chen , et al. 2024. “Study on the Mechanism of Lactic Acid Bacteria and Their Fermentation Broth in Alleviating Hyperuricemia Based on Metabolomics and Gut Microbiota.” Frontiers in Nutrition 11: 1495346. 10.3389/fnut.2024.1495346. 39698246 PMC11652139

[fsn370982-bib-0104] Rodriguez, J. M. , M. Garranzo , J. Segura , et al. 2023. “A Randomized Pilot Trial Assessing the Reduction of Gout Episodes in Hyperuricemic Patients by Oral Administration of *Ligilactobacillus salivarius* CECT 30632, a Strain With the Ability to Degrade Purines.” Frontiers in Microbiology 14: 1111652. 10.3389/fmicb.2023.1111652. 36865781 PMC9971985

[fsn370982-bib-0105] Salati, L. M. , C. J. Gross , L. M. Henderson , and D. A. Savaiano . 1984. “Absorption and Metabolism of Adenine, Adenosine‐5′‐Monophosphate, Adenosine and Hypoxanthine by the Isolated Vascularly Perfused Rat Small Intestine.” Journal of Nutrition 114, no. 4: 753–760. 10.1093/jn/114.4.753. 6716178

[fsn370982-bib-0106] Sautin, Y. Y. , and R. J. Johnson . 2008. “Uric Acid: The Oxidant‐Antioxidant Paradox.” Nucleosides, Nucleotides & Nucleic Acids 27, no. 6: 608–619. 10.1080/15257770802138558. PMC2895915 18600514

[fsn370982-bib-0107] Seth, A. , F. Yan , D. B. Polk , and R. K. Rao . 2008. “Probiotics Ameliorate the Hydrogen Peroxide‐Induced Epithelial Barrier Disruption by a PKC‐ and MAP Kinase‐Dependent Mechanism.” American Journal of Physiology. Gastrointestinal and Liver Physiology 294, no. 4: G1060–G1069. 10.1152/ajpgi.00202.2007. 18292183 PMC2653458

[fsn370982-bib-0108] Shan, R. Q. , Y. Ning , Y. Ma , et al. 2021. “Incidence and Risk Factors of Hyperuricemia Among 2.5 Million Chinese Adults During the Years 2017–2018.” International Journal of Environmental Research and Public Health 18, no. 5: 2360. 10.3390/ijerph18052360. 33671018 PMC7957707

[fsn370982-bib-0109] Shao, T. , L. Shao , H. Li , Z. Xie , Z. He , and C. Wen . 2017. “Combined Signature of the Fecal Microbiome and Metabolome in Patients With Gout.” Frontiers in Microbiology 8: 268. 10.3389/fmicb.2017.00268. 28270806 PMC5318445

[fsn370982-bib-0110] Shen, X. Y. , A. J. Xie , Z. J. Li , et al. 2024. “Research Progress for Probiotics Regulating Intestinal Flora to Improve Functional Dyspepsia: A Review.” Food 13, no. 1: 151. 10.3390/foods13010151. PMC10778471 38201179

[fsn370982-bib-0111] Sheng‐Nan, H. , L. Zhi‐Jian , Z. Bing , et al. 2015. Correlation Between Structural Shifts of Gut Microbiota and Hyperuricemia in Quails.

[fsn370982-bib-0112] Silva, J. C. P. , M. Mota , F. O. Martins , et al. 2018. “Intestinal Microbial and Metabolic Profiling of Mice Fed With High Glucose and High‐Fructose Diets.” Journal of Proteome Research 17, no. 8: 2880–2891. 10.1021/acs.jproteome.8b00354. 29923728

[fsn370982-bib-0113] Simic, M. G. , and S. V. Jovanovic . 1989. “Antioxidation Mechanisms of Uric Acid.” Journal of the American Chemical Society 111, no. 15: 5778–5782.

[fsn370982-bib-0114] Singh, A. K. , S. S. K. Durairajan , A. Iyaswamy , and L. L. Williams . 2024. “Elucidating the Role of Gut Microbiota Dysbiosis in Hyperuricemia and Gout: Insights and Therapeutic Strategies.” World Journal of Gastroenterology 30, no. 40: 4404–4410. 10.3748/wjg.v30.i40.4404. 39494101 PMC11525862

[fsn370982-bib-0115] Singh, J. A. , and J. D. Cleveland . 2019. “Comparative Effectiveness of Allopurinol and Febuxostat for the Risk of Atrial Fibrillation in the Elderly: A Propensity‐Matched Analysis of Medicare Claims Data.” European Heart Journal 40, no. 36: 3046–3054. 10.1093/eurheartj/ehz154. 30919894

[fsn370982-bib-0116] Softic, S. , M. K. Gupta , G. X. Wang , et al. 2018. “Divergent Effects of Glucose and Fructose on Hepatic Lipogenesis and Insulin Signaling.” Journal of Clinical Investigation 128, no. 3: 1199. 10.1172/jci99009. 29493547 PMC5824865

[fsn370982-bib-0117] Song, S. , Y. Lou , Y. Mao , et al. 2022. “Alteration of Gut Microbiome and Correlated Amino Acid Metabolism Contribute to Hyperuricemia and Th17‐Driven Inflammation in Uox‐KO Mice.” Frontiers in Immunology 13: 804306. 10.3389/fimmu.2022.804306. 35197978 PMC8858814

[fsn370982-bib-0118] Stewart, D. J. , V. Langlois , and D. Noone . 2019. “Hyperuricemia and Hypertension: Links and Risks.” Integrated Blood Pressure Control 12: 43–62. 10.2147/ibpc.S184685. 31920373 PMC6935283

[fsn370982-bib-0119] Stow, R. A. , and J. R. Bronk . 1993. “Purine Nucleoside Transport and Metabolism in Isolated Rat Jejunum.” Journal of Physiology 468: 311–324. 10.1113/jphysiol.1993.sp019773. 8254512 PMC1143828

[fsn370982-bib-0120] Sun, H. L. , Y. W. Wu , H. G. Bian , et al. 2021. “Function of Uric Acid Transporters and Their Inhibitors in Hyperuricaemia.” Frontiers in Pharmacology 12: 667753. 10.3389/fphar.2021.667753. 34335246 PMC8317579

[fsn370982-bib-0121] Sun, S. Z. , B. D. Flickinger , P. S. Williamson‐Hughes , and M. W. Empie . 2010. “Lack of Association Between Dietary Fructose and Hyperuricemia Risk in Adults.” Nutrition & Metabolism 7: 16. 10.1186/1743-7075-7-16. 20193069 PMC2842271

[fsn370982-bib-0122] Sun, Y. , X. Ge , X. Li , et al. 2020. “High‐Fat Diet Promotes Renal Injury by Inducing Oxidative Stress and Mitochondrial Dysfunction.” Cell Death & Disease 11, no. 10: 914. 10.1038/s41419-020-03122-4. 33099578 PMC7585574

[fsn370982-bib-0123] Tanner, C. , J. Boocock , E. A. Stahl , et al. 2017. “Population‐Specific Resequencing Associates the ATP‐Binding Cassette Subfamily C Member 4 Gene With Gout in New Zealand Maori and Pacific Men.” Arthritis and Rheumatology 69, no. 7: 1461–1469. 10.1002/art.40110. 28371506 PMC5984252

[fsn370982-bib-0124] Tin, A. , J. Marten , V. L. Halperin Kuhns , et al. 2019. “Target Genes, Variants, Tissues and Transcriptional Pathways Influencing Human Serum Urate Levels.” Nature Genetics 51, no. 10: 1459–1474. 10.1038/s41588-019-0504-x. 31578528 PMC6858555

[fsn370982-bib-0125] Tran, C. D. , D. M. Grice , B. Wade , et al. 2015. “Gut Permeability, Its Interaction With Gut Microflora and Effects on Metabolic Health Are Mediated by the Lymphatics System, Liver and Bile Acid.” Future Microbiology 10, no. 8: 1339–1353. 10.2217/fmb.15.54. 26234760

[fsn370982-bib-0126] Turner, J. R. 2009. “Intestinal Mucosal Barrier Function in Health and Disease.” Nature Reviews. Immunology 9, no. 11: 799–809. 10.1038/nri2653. 19855405

[fsn370982-bib-0127] Vareldzis, R. , A. Perez , and E. Reisin . 2024. “Hyperuricemia: An Intriguing Connection to Metabolic Syndrome, Diabetes, Kidney Disease, and Hypertension.” Current Hypertension Reports 26, no. 6: 237–245. 10.1007/s11906-024-01295-3. 38270791

[fsn370982-bib-0128] Vázquez‐Ávila, J. A. , M. Zetina‐Martínez , and J. Duarte‐Mote . 2018. “Hiperuricemia e Hipertensión Arterial Sistémica: ¿Cuál Es la Relación? [Hyperuricemia and Systemic Arterial Hypertension: What Is the Relationship?].” Medicina Interna De México 34, no. 2: 278–287. 10.24245/mim.v34i2.1613.

[fsn370982-bib-0129] Vedder, D. , W. Walrabenstein , M. Heslinga , et al. 2019. “Dietary Interventions for Gout and Effect on Cardiovascular Risk Factors: A Systematic Review.” Nutrients 11, no. 12: 2955. 10.3390/nu11122955. 31817107 PMC6950134

[fsn370982-bib-0130] Vitetta, L. , and G. Gobe . 2013. “Uremia and Chronic Kidney Disease: The Role of the Gut Microflora and Therapies With Pro‐ and Prebiotics.” Molecular Nutrition & Food Research 57, no. 5: 824–832. 10.1002/mnfr.201200714. 23450842

[fsn370982-bib-0131] Wang, H. N. , L. Mei , Y. Deng , et al. 2019. “ *Lactobacillus brevis* DM9218 Ameliorates Fructose‐Induced Hyperuricemia Through Inosine Degradation and Manipulation of Intestinal Dysbiosis.” Nutrition 62: 63–73. 10.1016/j.nut.2018.11.018. 30852460

[fsn370982-bib-0132] Wang, L. , and J. Ye . 2024. “Commentary: Gut Microbiota Reduce the Risk of Hyperuricemia and Gout in the Human Body.” Acta Pharmaceutica Sinica B 14, no. 1: 433–435. 10.1016/j.apsb.2023.11.013. 38261824 PMC10793086

[fsn370982-bib-0133] Wang, Q. W. , X. Wen , and J. M. Kong . 2020. “Recent Progress on Uric Acid Detection: A Review.” Critical Reviews in Analytical Chemistry 50, no. 4: 359–375. 10.1080/10408347.2019.1637711. 31296022

[fsn370982-bib-0134] Wang, Z. , Y. Li , W. Liao , et al. 2022. “Gut Microbiota Remodeling: A Promising Therapeutic Strategy to Confront Hyperuricemia and Gout.” Frontiers in Cellular and Infection Microbiology 12: 935723. 10.3389/fcimb.2022.935723. 36034697 PMC9399429

[fsn370982-bib-0135] Waring, W. S. 2002. “Uric Acid: An Important Antioxidant in Acute Ischaemic Stroke.” QJM: Monthly Journal of the Association of Physicians 95, no. 10: 691–693. 10.1093/qjmed/95.10.691. 12324642

[fsn370982-bib-0136] Waring, W. S. , D. J. Webb , and S. R. Maxwell . 2001. “Systemic Uric Acid Administration Increases Serum Antioxidant Capacity in Healthy Volunteers.” Journal of Cardiovascular Pharmacology 38, no. 3: 365–371. 10.1097/00005344-200109000-00005. 11486241

[fsn370982-bib-0137] Waring, W. S. , D. J. Webb , and M. Srj . 2000. “Effect of Local Hyperuricaemia on Endothelial Function in the Human Forearm Vascular Bed.” British Journal of Clinical Pharmacology 49: 511.

[fsn370982-bib-0138] Wei, W. , L. J. Zhou , S. Wang , et al. 2022. “ *Katsuwonus pelamis* Peptide and Its Complexes Protect Zebrafish and Mice From Hyperuricemia Through Promoting Kidney Excretion of Uric Acid and Inhibiting Liver Xanthine Oxidase Activity.” Frontiers in Chemistry 10: 924371. 10.3389/fchem.2022.924371. 35836673 PMC9273819

[fsn370982-bib-0139] Wilson, L. , and J. J. Saseen . 2016. “Gouty Arthritis: A Review of Acute Management and Prevention.” Pharmacotherapy 36, no. 8: 906–922. 10.1002/phar.1788. 27318031

[fsn370982-bib-0140] Winter, S. E. , and A. J. Baumler . 2023. “Gut Dysbiosis: Ecological Causes and Causative Effects on Human Disease.” Proceedings of the National Academy of Sciences of the United States of America 120, no. 50: e2316579120. 10.1073/pnas.2316579120. 38048456 PMC10722970

[fsn370982-bib-0141] Wrigley, R. , A. J. Phipps‐Green , R. K. Topless , et al. 2020. “Pleiotropic Effect of the ABCG2 Gene in Gout: Involvement in Serum Urate Levels and Progression From Hyperuricemia to Gout.” Arthritis Research & Therapy 22, no. 1: 45. 10.1186/s13075-020-2136-z. 32164793 PMC7069001

[fsn370982-bib-0142] Wu, Y. , Z. Ye , P. Y. Feng , et al. 2021. “ *Limosilactobacillus fermentum* JL‐3 Isolated From ‘Jiangshui’ Ameliorates Hyperuricemia by Degrading Uric Acid.” Gut Microbes 13, no. 1: 1–18. 10.1080/19490976.2021.1897211. PMC8007157 33764849

[fsn370982-bib-0143] Xiao, J. , X. L. Zhang , C. S. Fu , et al. 2015. “Soluble Uric Acid Increases NALP3 Inflammasome and Interleukin‐1β Expression in Human Primary Renal Proximal Tubule Epithelial Cells Through the Toll‐Like Receptor 4‐Mediated Pathway.” International Journal of Molecular Medicine 35, no. 5: 1347–1354. 10.3892/ijmm.2015.2148. 25813103

[fsn370982-bib-0144] Xie, A. J. , S. S. Zhao , Z. F. Liu , et al. 2023. “Polysaccharides, Proteins, and Their Complex as Microencapsulation Carriers for Delivery of Probiotics: A Review on Carrier Types and Encapsulation Techniques.” International Journal of Biological Macromolecules 242: 124784. 10.1016/j.ijbiomac.2023.124784. 37172705

[fsn370982-bib-0145] Xie, W. R. , X. Y. Yang , Z. H. Deng , et al. 2022. “Effects of Washed Microbiota Transplantation on Serum Uric Acid Levels, Symptoms, and Intestinal Barrier Function in Patients With Acute and Recurrent Gout: A Pilot Study.” Digestive Diseases 40, no. 5: 684–690. 10.1159/000521273. 34872097

[fsn370982-bib-0146] Xiong, J. , D. Wen , H. Zhou , et al. 2022. “Occurrence of Aflatoxin M1 in Yogurt and Milk in Central‐Eastern China and the Risk of Exposure in Milk Consumers.” Food Control 137: 108928. 10.1016/j.foodcont.2022.108928.

[fsn370982-bib-0147] Xu, D. X. , Q. L. Lv , X. F. Wang , et al. 2019. “Hyperuricemia Is Associated With Impaired Intestinal Permeability in Mice.” American Journal of Physiology. Gastrointestinal and Liver Physiology 317, no. 4: G484–G492. 10.1152/ajpgi.00151.2019. 31369290

[fsn370982-bib-0148] Xu, M. S. , P. J. Tian , H. Y. Zhu , et al. 2022. “ *Lactobacillus paracasei* CCFM1229 and *Lactobacillus rhamnosus* CCFM1228 Alleviated Depression‐ and Anxiety‐Related Symptoms of Chronic Stress‐Induced Depression in Mice by Regulating Xanthine Oxidase Activity in the Brain.” Nutrients 14, no. 6: 1294. 10.3390/nu14061294. 35334950 PMC8953819

[fsn370982-bib-0149] Yamada, K. A. , and I. W. Sherman . 1981. “Purine Metabolism by the Avian Malarial Parasite Plasmodium Lophurae.” Molecular and Biochemical Parasitology 3, no. 4: 253–264. 10.1016/0166-6851(81)90056-6. 7278883

[fsn370982-bib-0150] Yamada, N. , C. Iwamoto , H. Kano , et al. 2016. “Evaluation of Purine Utilization by *Lactobacillus gasseri* Strains With Potential to Decrease the Absorption of Food‐Derived Purines in the Human Intestine.” Nucleosides, Nucleotides & Nucleic Acids 35, no. 10–12: 670–676. 10.1080/15257770.2015.1125000. 27906630

[fsn370982-bib-0151] Yamada, N. , C. Saito , Y. Murayama‐Chiba , H. Kano , Y. Asami , and H. Itoh . 2018. “ *Lactobacillus gasseri* PA‐3 Utilizes the Purines GMP and Guanosine and Decreases Their Absorption in Rats.” Nucleosides, Nucleotides & Nucleic Acids 37, no. 5: 307–315. 10.1080/15257770.2018.1454949. 29723107

[fsn370982-bib-0152] Yamada, N. , C. Saito‐Iwamoto , M. Nakamura , et al. 2017. “ *Lactobacillus gasseri* PA‐3 Uses the Purines IMP, Inosine and Hypoxanthine and Reduces Their Absorption in Rats.” Microorganisms 5, no. 1: 10. 10.3390/microorganisms5010010. 28282902 PMC5374387

[fsn370982-bib-0153] Yamanaka, H. 2011. “Japanese Guideline for the Management of Hyperuricemia and Gout: Second Edition.” Nucleosides, Nucleotides & Nucleic Acids 30, no. 12: 1018–1029. 10.1080/15257770.2011.596496. 22132951

[fsn370982-bib-0154] Yamanaka, H. , A. Taniguchi , H. Tsuboi , H. Kano , and Y. Asami . 2019. “Hypouricaemic Effects of Yoghurt Containing *Lactobacillus gasseri* PA‐3 in Patients With Hyperuricaemia and/or Gout: A Randomised, Double‐Blind, Placebo‐Controlled Study.” Modern Rheumatology 29, no. 1: 146–150. 10.1080/14397595.2018.1442183. 29446654

[fsn370982-bib-0155] Yan, Y. , B. Li , Q. Gao , et al. 2025. “Intestine‐Decipher Engineered Capsules Protect Against Sepsis‐Induced Intestinal Injury via Broad‐Spectrum Anti‐Inflammation and Parthanatos Inhibition.” Advanced Science 12, no. 10: 2412799. 10.1002/advs.202412799. 39836501 PMC11904959

[fsn370982-bib-0156] Yanai, H. , H. Adachi , M. Hakoshima , and H. Katsuyama . 2021. “Molecular Biological and Clinical Understanding of the Pathophysiology and Treatments of Hyperuricemia and Its Association With Metabolic Syndrome, Cardiovascular Diseases and Chronic Kidney Disease.” International Journal of Molecular Sciences 22, no. 17: 9221. 10.3390/ijms22179221. 34502127 PMC8431537

[fsn370982-bib-0157] Yang, C. , Q. He , Z. Chen , et al. 2022. “A Bidirectional Relationship Between Hyperuricemia and Metabolic Dysfunction‐Associated Fatty Liver Disease.” Frontiers in Endocrinology 13: 821689. 10.3389/fendo.2022.821689. 35250880 PMC8889101

[fsn370982-bib-0158] Yang, Y. , J. Tian , C. Zeng , et al. 2017. “Relationship Between Hyperuricemia and Risk of Coronary Heart Disease in a Middle‐Aged and Elderly Chinese Population.” Journal of International Medical Research 45, no. 1: 254–260. 10.1177/0300060516673923. 28222629 PMC5536609

[fsn370982-bib-0159] Yano, H. , Y. Tamura , K. Kobayashi , M. Tanemoto , and S. Uchida . 2014. “Uric Acid Transporter ABCG2 Is Increased in the Intestine of the 5/6 Nephrectomy Rat Model of Chronic Kidney Disease.” Clinical and Experimental Nephrology 18, no. 1: 50–55. 10.1007/s10157-013-0806-8. 23584883

[fsn370982-bib-0160] Yasiri, A. , and S. Seubsasana . 2020. “Isolation of Bile Salt Hydrolase and Uricase Producing *Lactobacillus brevis* SF121 From Pak Sian Dong (Fermented Spider Plant) for Using as Probiotics.” Journal of Pure and Applied Microbiology 14, no. 3: 1715–1722.

[fsn370982-bib-0161] Yerlikaya, A. , T. Dagel , C. King , et al. 2017. “Dietary and Commercialized Fructose: Sweet or Sour?” International Urology and Nephrology 49, no. 9: 1699. 10.1007/s11255-017-1579-x. 28382575

[fsn370982-bib-0162] Yin, H. , N. Liu , and J. Chen . 2022. “The Role of the Intestine in the Development of Hyperuricemia.” Frontiers in Immunology 13: 845684. 10.3389/fimmu.2022.845684. 35281005 PMC8907525

[fsn370982-bib-0163] Yoon, H. S. , J. H. Ju , J. E. Lee , et al. 2013. “The Probiotic *Lactobacillus rhamnosus* BFE5264 and *Lactobacillus plantarum* NR74 Promote Cholesterol Efflux and Suppress Inflammation in THP‐1 Cells.” Journal of the Science of Food and Agriculture 93, no. 4: 781–787. 10.1002/jsfa.5797. 22806829

[fsn370982-bib-0164] Yu, Y. R. , Q. P. Liu , H. C. Li , C. P. Wen , and Z. X. He . 2018. “Alterations of the Gut Microbiome Associated With the Treatment o f Hyperuricaemia in Male Rats.” Frontiers in Microbiology 9: 2233. 10.3389/fmicb.2018.02233. 30283432 PMC6156441

[fsn370982-bib-0165] Yu, Z. P. , J. Lowndes , and J. Rippe . 2013. “High‐Fructose Corn Syrup and Sucrose Have Equivalent Effects on Energy‐Regulating Hormones at Normal Human Consumption Levels.” Nutrition Research 33, no. 12: 1043–1052. 10.1016/j.nutres.2013.07.020. 24267044

[fsn370982-bib-0166] Yuan, X. , R. M. Chen , Y. Zhang , X. Q. Lin , and X. H. Yang . 2022. “Altered Gut Microbiota in Children With Hyperuricemia.” Frontiers in Endocrinology 13: 848715. 10.3389/fendo.2022.848715. 35574004 PMC9091909

[fsn370982-bib-0167] Zeng, J. , Y. Li , Y. Zou , Y. Yang , T. Yang , and Y. Zhou . 2024. “Intestinal Toxicity Alleviation and Efficacy Potentiation Through Therapeutic Administration of *Lactobacillus paracasei* GY‐1 in the Treatment of Gout Flares With Colchicine.” Food & Function 15, no. 3: 1671–1688. 10.1039/D3FO04858F. 38251779

[fsn370982-bib-0168] Zhang, C. W. , L. J. Li , Y. P. Zhang , and C. C. Zeng . 2020. “Recent Advances in Fructose Intake and Risk of Hyperuricemia.” Biomedicine & Pharmacotherapy 131: 110795. 10.1016/j.biopha.2020.110795. 33152951

[fsn370982-bib-0169] Zhang, L. H. , J. X. Liu , T. Jin , N. B. Qin , X. M. Ren , and X. D. Xia . 2022. “Live and Pasteurized *Akkermansia muciniphila* Attenuate Hyperuricemia in Mice Through Modulating Uric Acid Metabolism, Inflammation, and Gut Microbiota.” Food & Function 13, no. 23: 12412–12425. 10.1039/d2fo02702j. 36374311

[fsn370982-bib-0170] Zhang, T. J. , S. S. Bian , Y. Q. Gu , et al. 2020. “Sugar‐Containing Carbonated Beverages Consumption Is Associated With Hyperuricemia in General Adults: A Cross‐Sectional Study.” Nutrition, Metabolism, and Cardiovascular Diseases 30, no. 10: 1645–1652. 10.1016/j.numecd.2020.05.022. 32669242

[fsn370982-bib-0171] Zhang, X. , B. B. Mass , V. Talevi , R. Hou , K. E. North , and V. S. Voruganti . 2022. “Novel Insights Into the Effects of Genetic Variants on Serum Urate Response to an Acute Fructose Challenge: A Pilot Study.” Nutrients 14, no. 19: 4030. 10.3390/nu14194030. 36235682 PMC9570712

[fsn370982-bib-0172] Zhang, X. M. , Q. Nie , Z. M. Zhang , et al. 2021. “Resveratrol Affects the Expression of Uric Acid Transporter by Improving Inflammation.” Molecular Medicine Reports 24, no. 2: 564. 10.3892/mmr.2021.12203. 34109437 PMC8201466

[fsn370982-bib-0173] Zhao, F. , N. Tie , L. Y. Kwok , et al. 2024. “Baseline Gut Microbiome as a Predictive Biomarker of Response to Probiotic Adjuvant Treatment in Gout Management.” Pharmacological Research 209: 107445. 10.1016/j.phrs.2024.107445. 39396767

[fsn370982-bib-0174] Zhao, H. Y. , X. Y. Chen , L. Zhang , et al. 2022. “ *Lacticaseibacillus rhamnosus* Fmb14 Prevents Purine Induced Hyperuricemia and Alleviate Renal Fibrosis Through Gut‐Kidney Axis.” Pharmacological Research 182: 106350. 10.1016/j.phrs.2022.106350. 35843568

[fsn370982-bib-0175] Zhao, Z. A. , Y. Jiang , Y. Y. Chen , et al. 2022. “CDER167, a Dual Inhibitor of URAT1 and GLUT9, Is a Novel and Potent Uricosuric Candidate for the Treatment of Hyperuricemia.” Acta Pharmacologica Sinica 43, no. 1: 121–132. 10.1038/s41401-021-00640-5. 33767379 PMC8724292

[fsn370982-bib-0176] Zhen, H. , and F. Gui . 2017. “The Role of Hyperuricemia on Vascular Endothelium Dysfunction.” Biomedical Reports 7, no. 4: 325–330. 10.3892/br.2017.966. 28928970 PMC5590038

[fsn370982-bib-0177] Zhu, J. Y. , Y. L. Wang , Y. H. Chen , X. X. Li , Z. D. Yang , and H. Li . 2020. “Association Between Hyperuricemia, Gout, Urate Lowering Therapy, and Osteoarthritis A Protocol for a Systematic Review and Meta‐Analysis.” Medicine 99, no. 33: e21610. 10.1097/md.0000000000021610. 32872016 PMC7437763

[fsn370982-bib-0178] Zhu, Z. , Y. Gu , C. Zeng , et al. 2022. “Olanzapine‐Induced Lipid Disturbances: A Potential Mechanism Through the Gut Microbiota‐Brain Axis.” Frontiers in Pharmacology 13: 897926. 10.3389/fphar.2022.897926. 35991866 PMC9388751

[fsn370982-bib-0179] Zmora, N. , J. Suez , and E. Elinav . 2019. “You Are What You Eat: Diet, Health and the Gut Microbiota.” Nature Reviews Gastroenterology & Hepatology 16, no. 1: 35–56. 10.1038/s41575-018-0061-2. 30262901

